# Turn-Key Protocols for Food Safety Culture Improvement: A Narrative on Theory and Best Practice

**DOI:** 10.3390/foods15142540

**Published:** 2026-07-17

**Authors:** Ryk Lues, Juanita Jonker, Monique Visser, Namhla Skweyiya

**Affiliations:** Centre of Applied Food Sustainability and Biotechnology, Department of Life Sciences, Faculty of Health and Environmental Sciences, Central University of Technology, Bloemfontein 9301, Free State, South Africa; jjonker@cut.ac.za (J.J.); monique.visser@mxns.com (M.V.); namhlaskweyiya@woolworths.co.za (N.S.)

**Keywords:** food safety culture, behavioural factors, assessment, triangulation, improvement

## Abstract

Food safety culture (FSC) is a structured, measurable and enforceable element of the food industry, which is essential for building consumer trust and safeguarding consumer wellness. Regardless of the size of the organisation, FSC plays a pivotal role in safety and quality assurance and should be embedded in the company’s values and beliefs, ultimately manifesting in employee behaviours. FSC’s principal denominators encompass the fundamental principles of leadership, knowledge, engagement, environment, performance, and outcome. These principles collectively form a holistic framework that is pivotal for enhancing the efficacy and efficiency of safety initiatives in the food supply chain. FSC should be integrated into existing food safety standards, aligning with management systems and enjoying support and ownership at all levels and portfolios. Attaining a strong FSC requires commitment and active participation, not only from departments and sections directly involved with food handling, but also from administrative departments and branches. A non-conducive or weak culture, on the other hand, creates barriers to the achievement of safety goals and creates environments that may lead to product safety failures and non-conformances, with potential detrimental impacts on both consumer and business well-being. The absence of a specific culture constitutes a culture in itself, and, therefore, regular, valid, and reliable assessment is crucial for understanding the current state of FSC, without drawing generalised, superficial, or biased conclusions. In this study, the history and context of FSC are discussed, as well as narratives on assessment protocols and improvement initiatives. A trustworthy and ethical assessment is the first step in a three-phase process, involving assessment, alignment, and intervention to improve FSC, constituting various subcategories. The ultimate intent of this study is to provide turn-key solutions through presenting ideas, debating concepts and proposing interventions to guide and inform FSC improvement, culminating in safe and wholesome products.

## 1. Introduction

Rarely has a social science principle manifested more tangibly within the food industry than the well-known Peter Drucker quote: “Culture Eats Strategy for Breakfast”. The dichotomy between the human behavioural aspect, which has habitually been regarded as an inferior determinant of quality and safety compared with systems, standards and audits, has emerged as a fundamental factor in global food safety. This has gathered momentum with increases in food safety incidents in environments that were regarded as fully compliant. Hence, recent FSC principles have increasingly embraced human constructs, such as the values, beliefs, and behaviours that shape an organisation’s commitment to food safety [1], and ultimately, such principles have manifested in management systems (FSMSs), regulatory developments, and international benchmarking frameworks, such as GFSI, Codex Alimentarius updates, and ISO-based standards [1,2,3,4]. FSC alludes to a mindset where every individual in the organisation recognises his/her role in ensuring the safety of the food being produced or handled. FSC endeavours to recognise that compliance alone is insufficient, while the behaviours and attitudes of individuals within an organisation are also critical for preventing food safety incidents.

While FSC has become more prevalent in food management systems, a clear and operationalised understanding of how to assess, align, and systematically improve FSC is limited. Currently, the literature mainly focuses on conceptual definitions or retrospective analyses of failures resulting in a weak culture. Contrastingly, fewer studies report on structured, practice-oriented protocols for implementation across diverse contexts. Evidence linking FSC maturity to measurable organisational outcomes remains fragmented; therefore, a need exists for integrative approaches that combine behavioural insights with practical methodologies to guide consistent FSC improvement.

The current review follows a narrative approach and discusses concepts and protocols to guide and inform the processes pertaining to FSC. The methodology synthesises the existing literature and presents best practices to identify broader themes and current consensus, and reflects on case study experiences related to the implementation of FSC interventions in various industries. The information presented has also been synthesised from experiences and lessons learnt during several consultative FSC interventions by the authors in various industries, including the condiments, beverage, retail, dairy, fisheries, farming, chicken, fruit juice, canning, and baking sectors. This manuscript is focused on providing an all-in-one foundation for standardisation, decision-making, and interventions within safety and quality assurance portfolios in the food industry, as well as regulators and assessors across the farm-to-fork continuum. The ultimate applicability of this study lies in bridging the gap between conceptual understanding and practical application by integrating behavioural determinants, assessment methodologies, and implementation strategies into a coherent FSC improvement protocol.

In order to address the objectives of this review, the following topics will be focused on: determinants of behaviour that shape FSC; the evolution of FSC as a discipline and standard; FSC as an overarching versus complementary concept; FSC solution pipelines, methodologies and best practices; identifying and implementing interventions; auditing and assessment best practices; and contemporary and emerging technologies for continuous improvement.

## 2. Food Safety Culture Determinants

Similar to other industries, it can be deduced that the behaviour of individuals and groups within a food production organisation shapes its organisational culture. As a result, there exists a relationship between the behavioural determinants that influence how individuals and teams approach food safety practices [5]. Often, the primary behavioural determinant and motivator for food handlers to comply with food safety management systems (FSMSs) is the fear of retribution. This is rooted in the expectation that a breach of food safety protocols may lead to consequences, i.e., anything from a reprimand to customer complaints, audit failures, removal from supplier lists, or even a product recall. This raises the question of whether fear is an effective long-term strategy for ensuring compliance, and whether it is unrealistic to expect food handlers to autonomously embrace food safety practices without ongoing monitoring and corrective measures. Fear is just one of many factors that shape human behaviour, being complex and multifaceted, shaped by a myriad of factors. Understanding these determinants is crucial for the field of FSC, especially since they assist in clarifying why food handlers think, feel, and act the way they do in different situations. The literature identifies numerous theories that explore determinants of behaviour that influence employee actions, including individual characteristics, as well as group dynamics and organisational culture [6].

### 2.1. Individual Characteristics

Personal traits play a fundamental role in shaping the behaviour of an individual within an organisation. In turn, individual characteristics influence outcomes such as productivity, job satisfaction, turnover, and absenteeism [7]. Individual characteristics further affect FSC and influence how employees perceive, understand, and adhere to safety protocols. The following fundamental individual characteristics will be highlighted within the context of FSC: biological, psychological, social, and environmental factors.

Human behaviour across all levels of employees in the food industry, ranging from floor workers to management, is fundamentally shaped by an intricate interplay of biological, psychological, social, and environmental factors. Biologically, physical elements like genetic inheritance, brain function, and hormonal influences underpin workplace actions [8]. For instance, genetic predispositions toward risk-taking or aggression can impact adherence to strict food safety protocols, *vis-à-vis* competitiveness or team-bonding during high-pressure shifts. Complementing this, psychological factors, such as personality, perception, cognition, emotions, and past experiences, which are vital to behavioural development within the food sector [7], dictate how personnel react to their environment. These psychological responses are often learned through consequence, particularly during developmental phases [9], where explicit recognition for maintaining hygiene standardises a strong work ethic, whereas line-management authority may cultivate workplace anxiety or fear of consequences, especially in terms of reporting safety hazards. Social, behavioural, and identity formation are heavily driven by cultural, peer or collegial validation, family dynamics, and organisational socialisation, through which employees internalise values and cultures [8]. These social behaviours are directed by cultural norms, where staff from diverse backgrounds and nationalities may prioritise group harmony and cooperation. Macro-environmental factors like socioeconomic status also affect employee behaviour, as workers from disadvantaged backgrounds, who are frequently employed in the food sector, are likely to experience challenges that can manifest as workplace hostility or disengagement, compared with higher income groups, who prioritise stability and security [10]. These behavioural outcomes are further shaped by employees’ domestic environments, for example, in densely populated, high-stress urban settings with crime, transport, and logistical limitations, compared with isolated, rural environments with limited healthcare access [10]. Broader societal prejudices related to, for example, race, gender, and religion, are further likely to impact how employees engage with their organisational dynamics and culture [11].

### 2.2. Group Theories and Dynamics

**Figure 1:**
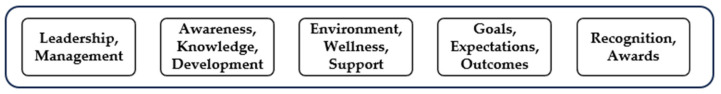
Conceptual illustration of overarching employee behavioural determinants influencing organisations’ FSC (authors’ summary).

In industrial environments, employee behaviour is not only affected by individual characteristics but also by the behaviour of co-workers. This is significantly augmented by the complex and often high-demand environments in the food sector, including lengthy shifts, protracted processing times, significant product volumes, and workload demands. Interpersonal behaviour can be mutually cooperative or conflicting, the former occurring where complementary transactions take place accompanied by behaviours such as mutual trust and respect. Conflicting behaviour occurs, on the other hand, where there are different or conflicting personalities or value systems [7]. In an environment where group dynamics are important, the interpersonal behaviour of the group also determines the behaviour of individuals. Hence, within the food safety context, group dynamics may influence the extent to which employees adhere to safety regulations and communicate about issues (e.g., contamination or hazardous practices). It may also influence the manner in which employees provide mutual support in maintaining the safety of a product. Within a particular production facility, group dynamics, such as leadership, training, and employee support systems, comprise behavioural determinants (Figure 1) which are critical in fostering a positive FSC.

Leadership and management style, in particular, influence how food-related policies are communicated and enforced [12]. Food handlers who feel well-supported and empowered through training and development opportunities are more likely to buy into the organisational food safety standards and values. An environment that prioritises employee well-being has been shown to enhance cooperation, improve morale, and encourage proactive engagement with food safety practices. Clear goals and recognition systems assist with the alignment of individual behaviour with organisational values, ensuring a consistent approach to food safety across the board. Several authors in the field of organisational culture have categorised these mentioned factors influencing behaviour into further constructs that are articulated through biological, physiological, social, and environmental factors, providing enablers to address such factors in the workplace, and thereby have proposed tangible interventions addressing such behavioural influences [1,13,14,15].

The definition of FSC and the principle of shared culture are grounded in ‘Systems Theory’, which suggests that human beings have an inherent desire to belong to a collection of like-minded individuals working toward common goals [16]. Therefore, a well-established organisational culture extends beyond the capacity of formal FSMSs within the food safety context. Additionally, organisational culture has the benefit of being easily imparted and sustained through resources such as communication, leadership, and shared values, instead of mere financial incentives.

Food safety and quality departments, especially in larger organisations, often experience difficulty securing financial resources to manage food safety, as well as the necessary time and attention to adequately address food safety concerns. This difficulty is augmented by prevailing economic pressures that often compel management to prioritise turnover and financial performance over product integrity. For quite some time, organisational leadership in the food sector has focused mainly on complying with legal requirements, as this was deemed the only relevant measure to mitigate regulatory risk [17]. In order to elevate food safety to a higher priority in organisations, it should be seen as more than a mere legal requirement, as it ultimately contributes to production and thereby contributes to fiscal well-being. Recent developments have shown that safety, or the lack thereof, can play a fundamental part in organisational prosperity or demise [15,18,19]. It should be reiterated that food safety culture should not be promoted “instead of” compliance and systems, but “in addition to” or “beyond”. Therefore, once food safety compliance has been met through the implementation and management of proper food safety assurance, the importance of promoting an organisational philosophy that contributes to an effective and conducive FSC is pivotal in taking food safety performance to the next level. A conducive culture is not only beneficial in terms of its contribution to food safety and risk mitigation, but also benefits organisational advancement, diligence, productivity, and compliance.

Although the body of knowledge on empirical evidence linking a robust FSC to compliance outcomes is still growing, tangible information remains limited in the wider farm-to-fork continuum. Case studies on significant foodborne illness outbreaks attributed to weak FSCs [20,21] are, however, mounting in the public domain. In these cases, organisational leaders have often overlooked food hygiene and prioritised cost savings over safety, leading to inadequate equipment maintenance and poor food safety practices. The findings conclude that employees in organisations with strong FSCs are generally more likely to adhere to food safety standards, which could lead to higher food quality and a reduced risk of foodborne illness outbreaks [20,22]. The body of knowledge on direct connections between a strong FSC and improved food safety, supported by unequivocal inferential indicators, is rapidly expanding, although it is still limited in some sectors. Four foundational dimensions (Section 2.2.1, Section 2.2.2, Section 2.2.3 and Section 2.2.4) have been identified through which organisational culture can shape employee behaviour to improve performance beyond compliance.

#### 2.2.1. Alignment with Food Safety Values and Goals

Organisational culture assists employees in internalising and aligning their behaviour with the company’s vision, mission, and core values [23,24,25]. Unlike other behavioural determinants, which may be influenced by personal motivations or external factors, a strong organisational culture is aligned with a shared purpose. This alignment motivates employees to pursue common goals. Organisational culture establishes broader social norms which govern acceptable behaviour, transcending other behavioural determinants outside of the occupational environment. Ambiguity is further reduced, the environment is transparent, and employees are clearly informed of expectations in various situations.

#### 2.2.2. Sustainability and Consistency

Consistency has often been highlighted in the FSC-related literature as an important determinant of behaviour, primarily as a result of its role in minimising uncertainty and ensuring stability. Culture plays a fundamental part in promoting consistency in employee behaviour, providing a long-term and flexible method for behavioural alignment rather than external motivators like financial incentives or punitive actions [19,23,26,27]. This is evident when cultural norms and values are integrated into daily practices. Such norms and values foster sustainable behaviour changes that persist over time. A well-defined organisational culture is easier to scale and replicate across different teams, departments, branches, or even geographies. A well-defined and repeatable FSC may, thus, provide a consistent set of expectations that can be communicated to new or re-deployed employees, ensuring that behaviours remain aligned with the organisation’s goals and values. As a result, this is more efficient than relying solely on individualised behavioural motivators, which can vary significantly across diverse teams.

#### 2.2.3. Food Handler Morale and Engagement

A strong and positive organisational culture prioritises a supportive and inclusive work environment [22,23,28]. Employee engagement is strongly enhanced by an environment where employees feel respected, valued, supported, and motivated. This results in employee morale and engagement often being more powerful than individual psychological motivators, as culture taps into collective social and emotional needs, making employees more committed and loyal to the organisation. A conducive organisational culture increases employee satisfaction by creating a work environment that fosters personal growth, inclusivity, and recognition. Employees who feel that they are a good fit within the organisation’s culture and “part of the team” are less likely to disengage compared with those motivated by external incentives or personal psychological drivers.

#### 2.2.4. Teamwork

While other behavioural determinants such as individual personality or cognitive biases may hinder collaboration, a culture that emphasises collaboration and collective success encourages behaviours that support teamwork [23,29]. Organisational culture provides a framework for addressing and resolving conflicts constructively. This suggests that a culture that emphasises respect, open communication, and problem-solving will drive behaviours that prioritise collaboration and peaceful resolution, even in stressful or high turnover situations. Such conflict resolution can often be more effective than relying on external authorities or individualised conflict resolution strategies. Additionally, a culture that encourages risk-taking, learning, and adaptation can drive behaviour that promotes innovation and flexibility [30,31]. While individual psychological traits like openness to experience also influence innovation, an organisational culture that prioritises continuous learning and creative problem-solving helps employees overcome fear of failure and embrace change.

## 3. The Emergence of FSC

Traditionally, food safety has been situated within the disciplines of microbiology and public health in terms of both its scientific and regulatory hosts [24,30,32]. As food science developed and with its increased awareness of consumer rights, an understanding emerged of the unique risks related to food processing and consumption [13]. Alongside the extensive movement of foods across borders, the concepts of food safety, food hygiene, nutrition, food process engineering, and environmental health have evolved. These concepts are intermingled into multiple multi-, inter-, and cross-disciplinary elements that ultimately ascertain the safety of specific food products. In order to ensure food safety, these elements have been incorporated into a few national and international standards and procedures that should ultimately be implemented, managed, monitored, and complied with [1,22]. As a result of this complex regulatory and scientific evolution, a growing recognition has emerged of the critical role that organisational and behavioural dimensions, now encapsulated in the concept of FSC, play in safeguarding food across the supply chain.

As a scientific niche, FSC has been developing steadily over the past approximately two decades, with a few scholarly publications emerging on the topic. Hence, certain prominent national standards have recently included FSC concepts, and independent consultants and training institutions providing services relating to FSC metrics and interventions have been steadily increasing [1,25,30]. In terms of the development of FSC, obvious parallels exist with the discipline of occupational health and safety (OHS) to the extent that OHS can probably be seen as the forerunner of the FSC concept [1,20]. Moreover, in recent years, FSC has received attention as a result of an increase in foodborne outbreaks globally, as well as accompanying litigation and convictions [18,19,30,33]. Ironically, in a number of these cases, the specific food provider may have complied with the regulations and standards stipulated, but failed to mitigate relevant hazards, thereby failing to demonstrate adequate preventive measures. Thus, this reiterates the principle that beyond compliance, an array of multi-faceted constructs exist that all interact to guarantee safe food products, and because of the integral role of the human component in food manufacturing, a conducive FSC remains a critical factor.

Although FSC has evolved into various models, constructs, and methodologies, the literature generally agrees that the key denominators comprising FSC include:

- Leadership: Committed leadership is essential for fostering FSC. Leaders must prioritise food safety, allocate resources, and set an example for the employees. The Food and Drug Administration (FDA) underscores the importance of leadership commitment to food safety [1,30,34].
- Knowledge and Awareness: Knowledge is a principal determinant of behaviour, and it speaks for itself that competency through effective training is fundamental in ensuring that food handlers grasp and implement food safety in their daily operations. In leading economies, such as the USA, the Food Safety and Modernisation Act (FSMA) in the United States emphasises the need for employee training in food safety [1,34].
- Communication: Open and transparent communication is vital for sharing not only information related to food safety incidents, performance, and improvements, but also related to the wider organisational context. A prominent voice promoting communication is the Food Standards Agency (FSA) in the UK, which reiterates the significance of communication in FSC [34,35].
- Commitment and Responsibility: The Codex Alimentarius Commission outlines the importance of accountability in food safety management [32]. Without assigned roles and responsibilities, employees may embrace the concepts, but not realise their respective responsibilities.
- Continuous Improvement: Regular assessment, feedback, and the implementation of corrective actions are essential for maintaining and enhancing FSC [3].

Questions may arise as to how the terminologies in Figure 1 relate to the FSC denominators alluded to above, as well as amongst the various terminologies referred to in the literature and materials used and promoted. Different models use particular overarching terms that are subcategorised into related sub-terms; for example, “leadership” may be regarded as an overarching theme with “engagement”, a sub-theme to be instilled through competent leadership, whilst in other scenarios engagement may be regarded as a sub-theme under “communication”. Apart from linguistic preferences and semantics informed by societal or organisational environments, the variations amongst FSC terminologies are also affected by licensing and IP considerations whereby registered standards, documents, and instruments are copyrighted, with the implication of alternative instruments being developed without infringing on the copyright, although still providing the needed FSC outcomes.

### 3.1. Scientific Progression

The scientific literature presents several proposed definitions to conceptualise FSC. The Global Food Safety Initiative (GFSI) defines FSC as “shared values, beliefs and norms that affect mindset and behaviour toward food safety in, across and throughout an organisation” [1]. The most frequently cited definition from Griffith et al. [36] is that FSC is “the aggregation of the prevailing, relatively constant, learned, shared attitudes, values and beliefs contributing to the hygienic behaviours used within a particular food handling environment.” Additionally, Yiannis [13] distinguished between FSC and FSMSs by noting that, while the implementation of an FSMS is essential, FSC extends beyond procedural frameworks to encompass human behaviour. This distinction is extensively acknowledged in the literature on FSC, emphasising the collective attitudes, values, and beliefs related to food safety that are jointly upheld by both employees and leadership within the organisation. Several studies suggest that evaluating FSC requires an all-inclusive approach, considering factors such as employees’ perceptions of food safety, leadership commitment, the sharing of knowledge, accountability, risk perception, and the overall work environment [13,24,36]. The mentioned conceptualisations highlight that science and academia endorse the notion that FSC is a multidimensional and transdisciplinary construct, influenced not only by individual awareness but also by organisational systems and social dynamics.

W. Edwards Deming, who has been widely acknowledged as the pioneer of total quality management, stated that a bad system will beat a good person every time [37]. This statement also manifests within the food safety framework, provided that engaged leaders in the food industry recognise that a strong culture is a crucial component of success [20]. As a result, these leaders prioritise the development and maintenance of an effective FSMS and a robust FSC, which includes rigorous safety measures embedded in fundamental business strategies [38]. FSC is intrinsically linked to organisational culture, which Schein [39] defined as “a set of broadly accepted fundamental beliefs and assumptions that a group forms while navigating challenges”. Thus, once these beliefs and assumptions prove trustworthy, they are collectively adopted by the entire group, serving as guiding principles for workers. Understanding these foundational FSC aspects is critical, given that they directly influence how food safety values are communicated, adopted, and maintained across organisations.

During the recent decade, FSC awareness has significantly evolved and is fundamental for ensuring food quality and reducing food-related risks across food supply chains. A strong FSC fosters consistent behaviour across organisational levels. As a result, when food safety values are aligned within the organisation, employees demonstrate higher compliance with protocols, prioritise food safety as part of ongoing work practices, and take initiative to minimise food safety hazards. This integration results in a reduction in food safety violations, decreased financial burden resulting from food recalls, and enhanced consumer trust [24]. The concept of FSC was expanded upon by Jespersen et al. [40], Nyarugwe et al. [39], and Sharman et al. [41] as an interplay of scientific and organisational elements of human behaviour. Therefore, the implementation of social cognitive theories, including risk perceptions, the illusion of control, and optimistic bias, is essential for establishing a deeper understanding of the human element [18]. Previous studies have identified the drivers of human behaviour and decision-making in the context of food safety frameworks [42]. The literature reflects on this human element by identifying the factors of leadership influence, cultural diversity, risk awareness, organisational commitment, and work environment [19,30]. Drawing from this behavioural perspective, the role of leadership becomes increasingly integral in incorporating these values across organisational processes.

Executive leadership, although having to carry loads of responsibilities much wider than only product safety, play an essential role in ensuring food safety compliance, not only from the perspective of performance, but also, ultimately, of accountability. The alignment of FSMSs with the organisation’s core objectives and values to uphold food safety goals across the entire organisation [13,43] is, therefore, not only a performance matter, but also a legal and economic risk mitigator. Leadership awareness of food safety principles and the FSMS portfolio is central to supporting evidence-based accountability, decision-making, and implementing measures that promote food safety across all levels [4]. Developing culture necessitates building trust through engaged leadership as an avenue to fostering employee commitment to food safety [43,44]. The synthesis of achieving organisational impacts serves as the cornerstone of effective food safety leadership [20].

Yiannas [45] echoed that while food safety managers function within a defined framework to manage FSMSs, effective food safety management requires strong overarching and executive leadership to provide impetus to preventative standards, drive implementation, and respond to crises [46]. In practice, leadership commitment involves participation in activities such as management review meetings, the implementation of food safety initiatives, active involvement during audits to determine the organisation’s current compliance status, and the implementation of required procedures. Leadership and management should not only cultivate a culture of responsibility and accountability, but also set an example across the organisation to foster an organisational culture [47]. To strengthen executive governance involves embedding values that “it is not just a job; it is a shared commitment” [1,48].

The advantage of FSC interventions is that such interventions are expected to have secondary benefits for the organisation, beyond only safety and compliance. Tappura et al. [49] mentioned the benefits of organisational culture as a secondary outcome beyond merely meeting compliance and certification requirements. The foundation of FSC is based on the understanding that the implementation of guidelines and protocols is insufficient. Rather, the objective is to create an environment within an organisation where every individual prioritises and practices food safety as a fundamental value. This concept started to emerge as research underscored the significance of organisational culture in ensuring food safety. Organisations are increasingly recognising the importance of fostering a collective commitment to safe practices, beyond just following regulations. Overall, the literature shaped by science and academia continuously highlights that FSC is an evolving paradigm—one that integrates structural, behavioural, and strategic elements to foster safer and more resilient food systems.

### 3.2. An Overarching or Complimentary Concept

Whether FSC is a ring-fenced component that runs parallel to food safety compliance, or an overarching concept that involves all components of food safety assurance, has been a point of debate. Various models and frameworks have been developed to assess and enhance FSC, which include criteria related to leadership commitment, communication, training, and employee engagement. Prominent models include the BRCGS FSC Excellence module, FSSC 22000’s integration with ISO 22000, and others tailored to specific industry needs [30,50]. Leaders widely acknowledge that the concepts that shape an organisation’s FSC focus on the following constructs:

- Shared responsibility: Everyone in the organisation, from top management to frontline workers, shares the responsibility for food safety.
- Open communication: Open communication about food safety concerns without fear of reprisal is crucial.
- Continuous improvement: A culture of continuous improvement fosters the adaptation and enhancement of food safety practices over time.

As presented in Table 1, the evolution of FSC standards highlights the growing acknowledgement and adoption of FSC into existing FSMSs, highlighting the demand for a harmonised and standardised approach across several sectors. Table 1 outlines the evolution of foundational FSC standards toward the latest version of FSSC 22000 (v6). The understanding of FSC has established global recognition, with organisations and standards bodies, including the Global Food Safety Initiative (GFSI), endorsing its importance [1]. Consequently, this recognition underscores the necessity for a reliable and standardised approach to FSC on a global scale. FSC has been progressively integrated into existing food safety standards, aligning with management systems such as ISO 22000, BRCGS, and FSSC 22000 [3,4,49]. This integration ensures that FSC is not a standalone concept but an essential part of overall food safety management. Irrespective of the shared elements, various segments of the food industry may have sector-specific challenges and expectations concerning FSC [51]. Therefore, frameworks and methodologies may be tailored to address sector-specific requirements, including food manufacturing, retail distribution, agriculture production, and food service operations.

During the last 10 to 15 years, industries such as food, consumer products, and transportation have increasingly recognised the crucial role of organisational culture in effectively implementing product safety management systems and preventing safety incidents. A strong safety culture has become well-established and is essential to ensure that employees consistently adhere to safety standards, even under pressure or when unsupervised. A weak culture not only puts barriers to the achievement of safety goals, but also wastes resources and creates conditions that may lead to serious product safety failures, with potentially devastating impacts on both consumers and the business itself [19,30,52].

The importance of fostering a positive product safety culture is acknowledged by major regulatory bodies, for instance Codex Alimentarius, Global Food Safety Initiative (GFSI), Brand Reputation Compliance Global Standards (BRCGS), and International Organisation for Standardisation (ISO), as well as national governments, and it has become a critical component of product safety audits [1,26,53]. Published empirical research emerged in 2008 and started to increase following the publication of the GFSI position paper in 2018 [1]. The GFSI recognised that technical compliance alone was not enough to ensure food safety across the food supply chain [1]. The GFSI further emphasised that FSC is essential regardless of the size of the organisation, and that food safety must be embedded in the organisation’s values, beliefs, and daily activities. The GFSI benchmarking requirements were updated in 2020 as a foundational reference to include FSC. The update necessitates that all GFSI-recognised Certification Programs and private standards (e.g., BRCGS, IFS, SQF, and FSSC 22000) include this requirement in their standards. The BRCGS Global Standard for Food Safety, Issue 8, was the first certification program to mandate an FSC requirement prior to the GFSI 2020 update. The requirement included a clear plan for developing and continuous improvement of food safety and quality culture [51].

In September 2020, the Codex Alimentarius Commission revised its global standard for general principles of food hygiene (CXC 1-1969) to include the concept of FSC. The Codex Alimentarius incorporated FSC under the topic “management commitment”, highlighting elements such as leadership, commitment, food hygiene awareness, and availability of sufficient resources for food hygiene systems [54]. The European Union adopted a similar approach in 2021, amending EU Regulation 2021/382 to integrate these new FSC requirements into Regulation (EC) No. 852/2004, the cornerstone of EU food safety legislation [20,30,49]. Describing that food business operators shall establish, maintain, and provide evidence of an adequate FSC by complying with the requirements regarding organisational commitment for safe food, leadership, food safety and hygiene standards, open communication, and the availability of adequate resources [55]. The Publicly Available Specification (PAS) 320 states that the framework supporting FSC is used for incorporating the FSC change plan into prevailing FSMSs [56]. The GFSI benchmarking requirements are aimed at aligning with the latest international standards and industry best practices. FSC elements were integrated into the fundamentals of FSMSs, demonstrating a framework to assess FSC as a compulsory requirement [57]. In its early stages, FSC was regarded as an abstract principle, i.e., a guiding philosophy rather than a measurable, enforceable standard.

Over time, there has been a movement toward developing FSC as a structured, measurable, and enforceable component within the food industry. The GFSI and its benchmarked standards (e.g., BRCGS and FSSC 22000) began incorporating elements designed to evaluate and measure an organisation’s FSC. These certifications recognise the role of cultural dimensions in safeguarding food safety. Additionally, the certifications include assessment criteria for leadership commitment, communication, training, and employee engagement designed to promote a strong FSC [1,28,30]. While Codex, GFSI, and related EU regulations share the fundamental goal of driving employee behavioural change from compliance to an internalised value system, they differ significantly in their legal weight and implementation mechanisms. The core similarity lies in their shared structural pillars: Codex Alimentarius, the Global Food Safety Initiative (GFSI), and European Union (EU) regulations all explicitly require management commitment, clear food safety goals, open workforce communication, and targeted employee training as the essential components of a robust food safety culture. The primary difference is found in their enforcement and scope: EU Regulation 2021/382 is a binding, statutory mandate, with legal penalties for food businesses operating within the European market. GFSI is a private, industry-driven benchmarking standard that requires highly specific, auditable performance metrics for commercial market access. Codex (specifically CXC 1-1969) acts as a non-binding, harmonised global advisory guideline that provides the foundational blueprint for both international trade and local legislative developments.

The industry is progressively moving from considering a strong FSC as a “nice to have” element to an essential requirement. As the importance of FSC continues to expand, there has been an upward shift in organisations regarding programs and strategies for the development, maintenance, and ongoing improvement of FSC [15,23,58]. This transition represents a significant change, transforming FSC from a theoretical concept to a practical, measurable, and essential component of the food industry. The progression from principles to certification in food safety management involves an in-depth exploration of various methodologies. As part of the transition from the foundational principles of food safety management to the practical setting of certification, it is crucial to consider the implementation methods, specifically science-based vs. behaviour-based FSMSs [1].

Table 2 provides an overview of the differences among the mentioned approaches, derived from various thought leaders [19,25,59,60] and industry standards [1,35], highlighting the unique characteristics in addressing the complexities of ensuring food safety. As organisations pursue certification, comprehending the implications of adopting either a science-based or behaviour-based system is essential. The presented comparative analysis serves as a valuable reference, aiding practitioners and researchers alike in understanding how these approaches can be integrated for the best FSC maturity in their specific contexts.

As both scientific-based and behaviour-based FSMSs play critical roles in ensuring the safety of food products, integrating the strengths of both approaches can create a comprehensive system. Such a comprehensive system will not only meet regulatory requirements but also foster a positive safety culture, driven by informed, responsible, and proactive employee behaviour. An illustrative case is the BRCGS framework, which incorporates a dedicated FSC Excellence module as an integral part of its standards, aiming to guide organisations in the assessment and enhancement of their FSC [51].

FSSC 22000 integrates FSC principles into its broader certification scheme, aligning with the ISO 22000 standard [4]. The current review explores the nuances of each standard’s approach, assessing the core components of each framework, integration strategies, assessment methods, and considerations for global recognition. A comparison of key aspects of FSC in BRCGS and FSSC 22000 is provided in Table 3 to facilitate a clearer understanding of the embedded FSC approaches. This comparative analysis may assist stakeholders in the food industry in making informed decisions regarding the adoption of standards that align with organisational goals and sector-specific requirements.

## 4. Best Practices and Lessons Learnt

The success of FSC initiatives is deeply rooted in data integrity and constructive engagement with all stakeholders. Another famous quote of Peter Drucker states: “If you can’t measure it, you can’t improve it” [61]. The essence of Drucker’s statement is clear: to improve any system, it is important to start with an evaluation of its current state, and this understanding requires accurate measurement. Measurement plays a critical role in assessing the efficiency of the current setting, recognising areas that need improvement, setting goals, and evaluating progress [1,15,25,28,49]. The ethical dimensions of assessing FSC should not be overlooked, as neglecting ethical principles can significantly harm both the organisation and the reputation of the FSC discipline [19,32,49]. Therefore, accurate and ethical assessment is the first step in a three-phase process of improving FSC involving assessment, alignment, and intervention. Each phase presents its own set of challenges and best-practice guidelines, but without an authentic and reliable assessment, subsequent efforts to improve FSC will be ineffective and potentially harmful.

In the context of FSC, measuring certain constructs, such as employee morale, leadership styles, or customer satisfaction, can be especially challenging [20,29,30]. As these constructs are often difficult to quantify, a combination of both qualitative and quantitative methods, for example, surveys, interviews, focus groups, and observational assessments, is necessary to gain a comprehensive understanding of the situation and drive improvement. In the global context, the task of assessing FSC becomes even more complex due to the many variances present across the farm-to-fork continuum. The variances are present between sectors, for instance, primary production, processing, transportation, wholesale, retail, food service, and catering, as well as in the informal food sector [18,20]. Compounding factors are the diversity of food types, varying regulations and standards, import dynamics, and the distinct cultural, belief, and regional differences present in the country [15,19,33]. In parallel, food safety, traditionally grounded in “hard sciences” such as microbiology and food engineering, has evolved to include human and behavioural sciences. This transition has enabled multidisciplinary engagement, integrating expertise from microbiology, engineering, consumer science, and management disciplines to address food safety using inclusive approaches.

**Figure 2:**
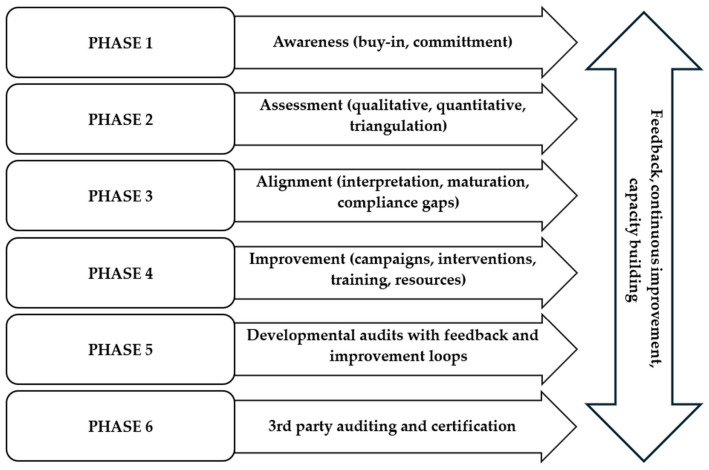
The FSC improvement pipeline conceptualised by Lues, encompassing the progression from awareness to compliance, incorporating feedback loops [62].

Integrating these diverse methodologies from different disciplines is not without its challenges, since using tools and techniques from disciplines outside one’s area of expertise can lead to flawed interpretations or ineffective interventions [26,49]. For example, applying the wrong approach to assessing FSC could result in misidentifying issues or misdirecting resources, potentially causing harm to organisational morale and leading to ineffective or even damaging interventions. Based on experiences and lessons learnt, this section of this review will explore best practices and lessons learnt in accurately and authentically assessing FSC to drive meaningful improvement. Suggesting a procedure responsive to the relevant requirements and intent of the standards may be quite daunting, but Figure 2 provides a clear pipeline (or model) for food companies in the farm-to-fork continuum to follow. The model allows for progression through the various constructs, responsive to the standards’ requirements, but flexible enough to provide leeway for industry to decide on the sections to target, informed by risks, logistics, and other considerations.

### 4.1. Phase 1: Creating Awareness and Buy-In

Achieving a strong FSC requires commitment and active participation from every level of the organisation. Leadership buy-in is crucial and starts with top-level management that visibly endorses and prioritises food safety through both resources and consistent messaging. It is equally important to engage employees at all levels, empowering the employees to contribute ideas and take ownership of food safety initiatives. Consistent communication, training, and defining clear objectives across the organisation are fundamental in the development of a culture that values and prioritises food safety [26,30].

The first phase of the process necessitates awareness and marketing strategies comprising presentations, discussions, and workshops on constructs and principles of FSC, its value, and impact. The activities may be conducted across various industry sectors, aligned with the initial audit requirements of major standards. The components include introductory and advanced FSC offerings (half and full day, respectively, in person or online), a 2-day FSC auditing course aligned with specific standards, and a train-the-trainer 3-day course on all of the components of FSC interventions to enable implementation by internal QC teams and consultants.

#### Acknowledging Territories

The involvement and support of departments and entities not directly involved with handling the product, such as sales, human resources, finance, and logistics, is of the utmost importance, and if not engaged diplomatically and discreetly, may pose consequences likely to derail the entire FSC intervention endeavour. The implementation of interventions that may overlap with responsibilities historically assigned to human resources (HR) creates the impression that HR has been negligent in performing its duty, or is subordinate to the FSC initiative [30]. This also pertains to external third-party contractors and consultants appointed to assist with broader industrial psychology and culture interventions. In most countries, professional registration in areas such as industrial psychology is required to ensure that consultants possess the necessary qualifications, ethical standards, and expertise to effectively assess and intervene in organisational culture projects [63]. Such professional registration maintains credibility, assures competence, and provides legal, ethical, and risk mitigation frameworks for accountability. Although FSC focuses on food safety, respondents are likely to respond and allude to concepts much wider than only the food safety narrative, raising sensitive issues like employee well-being, interpersonal incidents, and even perceived misconduct. Individuals and departments that are custodians of the human resource narrative may perceive FSC interventions as being led by unqualified individuals offering misleading or potentially harmful advice. In countries and environments where organised labour and union participation are prominent, FSC interventions may be seen as infringing on workers’ rights and autonomy. Depending on the dynamics and context, FSC interventions may be viewed as top-down efforts to control behaviours, attitudes, or values that affect how food handlers engage with their work environment.

Ultimately, the ability of the FSC team to ensure buy-in and support of all stakeholders through transparent processes, setting participants at ease, respecting authority, delegations, and the interests and voices of employees could limit possible tensions and ensure a valid and effective process.

### 4.2. Phase 2: Culture Assessment

All food companies already have a culture, meaning even a bad culture is a culture. Therefore, to approach the FSC process with a “once-size-fits-all” approach can not only be ineffective, but also demotivating and harmful. For example, an FSC intervention where the consultant recommends broad, all-inclusive training on components of ethical leadership, with line management that has already been exceptionally ethical and effective being instructed to attend alongside sections with poor and unethical leadership. This “what is good for the goose…” approach is likely to be quite demoralising to the individuals not warranting being in the particular training, implying that their best efforts are not being noted. Regular and comprehensive assessment is, therefore, vital to understanding the current state of FSC [60]. It is recommended to use a combination of assessment instruments like surveys, audits, interviews, and observations to gauge the existing culture. These assessments should cover various aspects, including employee attitudes, behaviours, leadership commitment, communication, and training effectiveness. The assessment instruments recommended are a combination of methodologies used and reported on in the literature [64], as well as adapted for application in several consultative projects. At the time of the current paper, the Food Focus firm was regarded as a leading service provider in FSC, with commentaries by stakeholders in the popular media relating to the assessment of best practices [65].

The second phase of the process involves the measurement of the existing FSC to inform further interventions. Ultimately, information from assessments is aimed at providing actionable insights, guiding organisational improvements by identifying areas needing intervention, and monitoring changes effectively. For instance, survey results revealing gaps in employee training can prompt targeted programs, which could assist with prioritising resources for critical areas, which in turn impacts FSC. Clear, data-driven insights foster continuous improvement, enabling organisations to refine practices based on empirical evidence [66].

Effective assessment processes entail various activities and triangulation, and are strongly informed by ethics, validity, and reliability. The assessment tools, quantitative and qualitative methodologies, and sampling and representation are pivotal in the process. The assessment process may be implemented in phases, based on the quality of content received via initial processes, the first being an online survey, supported by online or in-person interviews, focus group assessments, facility observations, and document analysis. The methodology, termed explanatory sequential design, includes combining quantitative and qualitative methods, where the quantitative phase precedes the qualitative phase in both data collection and analysis [67]. Following data collection, the effective processing, interpretation, and communication of findings are essential. Fundamentally, an understanding of descriptive and inferential statistics is required, as well as the integration of methodologies and disciplines. Implementing cognitive articulation toward tangible and effective outcomes, received from a variety of opinions and inputs, is pivotal.

#### 4.2.1. Best Practice Considerations

Although the concepts and practices alluded to in the following sections may be common knowledge in the domains of the social sciences and humanities, in the food production environment, the historical disciplines guiding food safety and quality have their roots in the biological sciences, such as food science, microbiology, public health, food technology, and the like. Practitioners in these disciplines may not be au fait in engaging with the social sciences, compromising validity and reliability and, in the worst cases, presenting potential risks in the domains of human well-being, legality, and harm. Therefore, considering the intent of the current study to present turn-key solutions, the inclusion of FSC best practices is essential. Considering that phase 2 encompasses contact with food handlers and staff of various levels, the observation of data collection good practice protocols is essential to mitigate risk but also ensure valid and reliable information, as outlined in the sections below.

(a) Reliability and Validity

Effective FSC assessments rely on reliable and valid data collection methods, which encompass methodological strategies, such as standardisation, triangulation, and statistical validation, all grounded in best practices and adapted to cultural and contextual diversity across the food industry. Reliable data collection methods refer to methods that are consistent (standardised) and accurate, while valid data collection methods truly measure what is intended to be measured [68]. The reliability and validity of the tools and methods used in FSC assessments are integral to the accuracy of the findings and the success of any interventions, and the chosen methodology must be grounded in established standards to ensure the results are meaningful and actionable.

Ensuring the reliability of FSC assessments is highly dependent on the use of standardised and consistent data collection instruments. Standardisation ensures that all respondents answer the same set of questions, promoting consistency, reliability, and data comparability. This uniformity is crucial for accurately assessing organisational culture, as it eliminates variability from different interpretations. Standardised surveys facilitate comparable data collection across different periods and departments, enabling organisations to track changes and trends effectively. Additionally, standardisation supports benchmarking, thus allowing comparison with industry standards or other entities, and streamlines the administration process [43].

An additional methodological approach in enhancing the validity and depth of FSC assessments is triangulation, which encompasses integrating a range of data sources and perspectives. The triangulation methodology, as it pertains to the context of FSC, describes multiple data sources, methods, or perspectives to achieve a broader, comprehensive and reliable understanding of an organisation’s culture. Triangulation contributes to ensuring that the findings are free from bias or excessive dependence on a single method or viewpoint, strengthening the validity and depth of the results [18,68]. Triangulation may involve the following:

- Collecting data from different groups within the organisation, such as employees at various levels (e.g., executives, managers, and front-line staff), or from different departments or teams. This supports the recognition of a more comprehensive understanding of the culture, as various elements of the organisation might experience and interpret the culture differently.
- Employing a combination of quantitative and qualitative methods. As an example, a survey with closed-ended questions (quantitative) may be supported by interviews or focus groups (qualitative), allowing for a deeper understanding of the cultural dynamics. The quantitative data provides measurable patterns, while qualitative data can offer deeper insights into the “why” behind those trends.
- Including different stakeholders in conducting the analysis, such as leadership, HR professionals, and frontline manufacturing staff. Each group may interpret cultural data through different lenses, which may highlight insights that may be overlooked from any limited perspective.

To ensure content validity, the formulation and review of constructs are often guided by input from academic and industry experts [13,38,39,69]. This process typically draws on established frameworks in the literature, while avoiding potential infringement on proprietary methodologies or copyrighted materials. The evolution of FSC as a certification standard further shapes the development of assessment themes and constructs, incorporating perspectives from global stakeholders and adhering to established best practice frameworks [1,4]. Commonly employed constructs include vision, inspiration, empowerment, performance, and change readiness, with adjustments made to reflect regional and cultural contexts and reduce bias. The refinement of survey content is frequently supported through iterative feedback sessions with domain specialists, contributing to enhanced construct clarity and contextual relevance. Such engagements also facilitate test-retest reliability assessments by allowing repeated evaluations across time and expert groups.

An academic–industry integrated approach offers the advantage of comparative analysis across various regions and sectors along the food supply chain, thereby enabling the continuous improvement of data collection instruments and enhancement of the relevance and applicability thereof. Considerations such as the use of clear, culturally appropriate language are central to instrument design, particularly in settings with diverse multicultural populations. Ensuring the validity and reliability of data collection methods is often approached as an evolving strategy, tailored to the linguistic and operational context of each food production or service environment. Strategies designed to uphold reliability and guide targeted interventions are generally informed by identified gaps rather than being guided by broad conceptual constructs. Interventions are typically aligned with thematic indicators, incorporating organisational priorities, risk factors, and recognised standards. As such, construct categorisation is often driven by semantic and linguistic associations rather than comprehensive conceptual models.

While Cronbach’s alpha is commonly applied to assess internal consistency in FSC assessments, its methodological limitations are well recognised. McDonald’s omega may also be considered to replace or supplement alpha, as in selected scenarios it provides a more accurate mathematical reflection of data reality. Challenges with the application of consistency determination include the presence of multidimensional constructs and non-normal data distributions, especially in smaller samples. In the food industry, the realities of workings shifts, survey dissonance, and disengagement often lead to challenges with response rates, causing skewed data. Additionally, variability in Cronbach’s alpha values may result from data translated into different languages, potentially affecting consistency. Furthermore, the evolving nature of FSC initiatives, as emphasised by standards such as FSSC 22000 (v6), may undermine the utility of Cronbach’s alpha for measuring test–retest reliability, as differing responses over time can reflect real organisational change rather than instrument inconsistency.

(b) Ethics and Authenticity

Ethical considerations are essential not only for risk mitigation, but also for ensuring the validity and reliability of the outcomes [68]. For instance, if a participant in a survey feels uncertain about the anonymity of their responses, he/she is unlikely to provide honest feedback. Thus, ethical principles must be in place to guarantee that assessments are both trustworthy and accurate. A vital principle to be observed at the onset of data collection is that FSC is an organisational endeavour and not only focused on a selected department. Hence, the participation of staff across levels of authority and specialist sections needs to feel included and grasp the principle that each member contributes to presenting a safe and wholesome product. The idea that, although sections and task descriptions may differ, all employees endeavour to present the final product to the end-user should be understood.

In some countries, additional laws and regulations direct the use of personal information, and it is essential that respondents submit consent for the use of personal information. For example, in South Africa, POPIA (Protection of Personal Information Act) is a law designed to protect the personal information of individuals. The Act regulates the collection, processing, storage, and sharing of personal data by organisations to ensure privacy and prevent misuse. Thus, the Act sets out conditions for lawful processing, including obtaining consent, ensuring data security, and allowing individuals to access and correct their personal data. The primary aim is to safeguard personal information while promoting responsible data handling practices by businesses and other entities [70]. The General Data Protection Regulation (GDPR)—European Union (EU), Data Protection Act of 2018 (United Kingdom), the Asia-Pacific Economic Cooperation (APEC) Privacy Framework (APEC Region), Australian Privacy Principles (APPs)—Australia and Personal Information Protection, and Electronic Documents Act (PIPEDA)—Canada are further examples of similar regulations.

(c) Informed consent and voluntary participation

Participants must be fully informed about the purpose, risks, and benefits of the assessment and must have the option to choose whether to participate. Therefore, participants should provide consent to partake in the data collection process. Participation should be entirely voluntary, with no perceived pressure. In certain contexts, this process involves engagement with organised labour, which may regard third-party interactions with their members as intrusive. It is crucial to build trust and goodwill by respecting the roles of internal stakeholders, such as HR departments, industrial psychologists, and union representatives. Also, the distinction between FSC and the broader organisational culture must be clearly communicated to avoid overstepping boundaries.

(d) Anonymity

Both quantitative and qualitative data collection methods should ensure respondent anonymity to encourage open and honest responses and to yield more accurate data, especially in cases of sensitive FSC topics. Anonymity mitigates retribution fears, fostering trust and obtaining authentic insights. This approach uncovers hidden issues, like hesitation to report food safety violations, essential for improving the overall FSC [71]. Protecting the anonymity and confidentiality of participants is particularly challenging in small sections or departments where responses might be easily traced back to individuals. However, aggregating data from small groups or using anonymous survey methods can help mitigate this issue [22,68]. The manner in which third-party assessors interact with employees should be carefully considered, promoting a sense of comfort and psychological safety amongst participants. Each assessment should avoid any perception of exposure or vulnerability and aim to minimise physical, emotional, or psychological harm to participants.

(e) Respecting culture and diversity

Different departments or locations may have distinct norms, values, and attitudes toward food safety. Acknowledging these contextual differences and adapting data collection methods and strategies to suit the unique characteristics of each segment within the organisation is important. Strategies that are effective in one department or location may not yield the same results in another due to aspects of diversity. Tailoring approaches to align with the existing cultural values of different departments or locations can significantly enhance the adoption of food safety practices [15,22]. Diverse workforces require an understanding of cultural, religious, and social values to ensure high participation rates and honest responses. The dynamics of addressing employees, particularly in cross-generational or hierarchical situations, must be carefully considered to avoid alienating participants or discouraging their input [19,20]. The way third-party assessors interact with staff also plays a critical role in fostering a positive and respectful environment.

(f) Objectivity

Entering a facility with different sections, line functions, portfolios, sub-facilities, and regions can be complex and, at times, overwhelming. This complexity necessitates acknowledgement that different sections have their own inherent cultures depending on the layout of the company. For example, production may have a different culture from packaging, which in turn may differ from logistics, etc. As previously mentioned, a one-size-fits-all approach is often ineffective and may even prove detrimental [49,72]. Therefore, utilising quantitative methodologies that allow for the identification of selected FSC shortcomings and facilitate room for improvement, in particular, for targeted areas, are essential. Empirical evidence suggests that the validity and reliability of FSC surveys are largely influenced by the sampling protocol and data collection instruments [60]. In large, complex organisations with diverse departments and cultural dynamics, a combination of stratified and purposive sampling techniques has proven effective. Stratified sampling enables the systematic division of the population into distinct subgroups [68,73], thereby ensuring that data is representative of the different cultural dynamics at play in each section of the organisation. Purposive sampling, on the other hand, facilitates the deliberate inclusion of role players or high-risk areas [68], ensuring that critical insights are captured from those most knowledgeable about or affected by FSC.

Considering that participation is both anonymous and voluntary, the only practical way to facilitate the identification of a particular area is by explicitly stating such in the survey document. This approach introduces its own sets of challenges, as respondents, particularly those from smaller sections, may fear potential identification. In these contexts, it is often possible for the reader of the report to relate a particular response in a given section to specific individuals. As a general guideline, when the stratum consists of fewer than ten individuals, it is advisable to aggregate data with other strata to protect anonymity. Additionally, some sources suggest avoiding the disclosure of section names when fewer than five individuals respond to a question, as this could increase the risk of re-identification. Another consideration in selecting the sampling protocol is the size and availability of the various selection strata, as well as logistical considerations such as size, locality, and resources. In a large organisation with multiple outlets and a broad respondent base, it is more practical to select specific areas to initiate the process based on criteria established in consultation with the organisation. Such sampling strategies may be informed by the following:

- Client certification due dates and requirements.
- Product risk portfolios.
- Previous compliance findings and complaints.
- Culture-related incidences and priorities.
- Locality and size of strata.
- Other logistical, seasonal, and respondent availability considerations.

(g) Inclusion criteria

During FSC interventions, a question that often arises (especially by support departments not directly involved with physical food handling) is whether FSC interventions should only be aimed at staff directly involved with product handling, or also encompass secondary management, administrative, and logistics sections. In the food sector, the idea exists that everyone, from leadership to cleaning staff, has an important role in achieving the shared goal of providing safe and wholesome food products to customers. Apart from being part of the team, the peripheral benefits of food safety being a shared value across all levels include being inspired, reflecting on the positives of the company outside the working environment, having the confidence to contribute to performance beyond one’s own portfolio, and ultimately contributing to risk mitigation. Ultimately, each employee in the company, irrespective of their position, has five senses that can be applied to identify and report hazards—it would be a huge loss if such employees opted not to apply their senses to this effect due to lacking confidence, feeling intimidated or disengaged, or excluded. Ironically, this will imply bypassing multiple “human monitoring systems” and having to rely only on traditional safety monitoring systems at considerable expense. Ultimately, a holistic approach to food safety is a shared responsibility across all levels and functions. Even non-food-handling staff, such as those in management, administration, HR, marketing, or logistics, are likely to influence food safety outcomes [18,33]. These staff’s actions can affect areas like reputation, training, communication, policy implementation, and maintenance, all of which contribute to a safer food production environment. Although front-line food handlers may follow food safety protocols, without support from other departments, the success of food safety initiatives can be compromised.

The participation in FSC interventions should extend beyond permanent food handlers and operational staff and include a broader range of individuals who interact with the food production environment. Therefore, consideration should be given to also include staff who have recently joined and may benefit from induction programs alluding to the importance of FSC. Temporary and seasonal workers, interns, consultants or commissioned contractors, and even visitors/groups may also be considered as encompassing the FSC intervention sample. Each of these groups presents distinct perspectives and levels of engagement, warranting tailored assessments and interventions that address their specific roles and influence within the organisational culture.

#### 4.2.2. Quantitative Data Collection

Quantitative methodologies entail collecting and analysing numerical data to identify patterns and averages, evaluate relationships, and make predictions applicable to broader populations. This approach is utilised across various disciplines to quantify opinions, attitudes, behaviours, and variables, employing tools, such as surveys, to gather data [74]. The goal is to determine whether variables have an impact on results within a specified sample. Quantitative methods are characterised by its systematic nature, collecting data from large samples that accurately represent the entire population. The results derived from the data are impartial, rational, and unbiased by the methodology’s design, which aims to eliminate any influence on the variables. The advantages of utilising quantitative assessments in data collection are discussed below.

(a) Impartiality

Quantitative surveys provide objective data that can be statistically analysed, reducing the subjectivity inherent in qualitative assessments. Thus, the surveys yield unbiased insights into organisational culture, essential for identifying areas needing improvement and making evidence-based decisions. For instance, Likert-scale questions can quantify employees’ perceptions of management’s commitment to food safety, leadership, communication, and employee engagement [39]. The aggregated data offers a holistic view of the organisation’s overall FSC [66]. Large sample sizes facilitate accurate generalisations, enhancing the credibility of the outcomes and minimising the impact of outliers [75].

In most food industries, depending on their size and structure, stratified and purposive sampling has been shown to be the most effective methodology, as these techniques accommodate representation amongst the various departments and units, accommodate targeted respondents in terms of authority and knowledge, and allow negotiating with logistical factors, such as shifts and availability. As mentioned earlier, such integrated sampling provides a more flexible, non-random approach, while it may introduce bias and limit generalisability.

(b) Scalability

Quantitative surveys can be administered to numerous participants across various departments, locations, and hierarchical levels, thus providing a comprehensive insight into organisational culture. The scalable nature of the approach makes it suitable for organisations of all sizes, enabling the collection of diverse perspectives and identification of common themes that might be overlooked in smaller samples. The ability to segment data also provides insights into specific subgroups within the organisation, ensuring inclusive and representative FSC assessments [24].

(c) Benchmarking

Quantitative surveys enable benchmarking against industry standards or historical data, helping organisations identify areas for improvement and measure the impact of interventions over time. Comparing survey results with industry benchmarks provides insights into relative performance in FSC, thereby highlighting areas of excellence or shortage. Historical comparisons allow for the assessment of progress over time, assessing whether initiatives aimed to improve FSC are yielding the desired results [43].

(d) Statistical analysis

Quantitative surveys facilitate rapid data collection and analysis using various statistical methods, such as correlation, regression, factor analysis, and structural equation modelling [74]. These statistical methods identify patterns and relationships within organisational culture, providing a deeper understanding of cultural dynamics. Additionally, statistical tools and software offer clear visualisations, such as charts and graphs, to interpret data, identify trends, and uncover relationships between organisational culture and food safety outcomes [38].

(e) Efficiency and cost-effectiveness

Quantitative surveys are highly effective in terms of time and resources, enabling organisations to collect large volumes of data quickly. These surveys may be integrated into standard assessment processes, providing ongoing monitoring of organisational culture without disrupting operational workflow. Such integration promotes comprehensive and sustainable cultural assessments over extended periods [24]. Quantitative surveys, especially when conducted online, are typically more cost-effective than qualitative methods, such as interviews and focus groups. They demand fewer resources and enable faster data collection. Importantly, this cost-effectiveness does not compromise data quality; rather, it enhances the capacity to gather comprehensive and reliable information within financial limitations [76].

(f) Measurement of change over time

Quantitative surveys are an effective tool for measuring changes in FSC over time, supporting the analysis of the effectiveness of interventions. This evolving perspective of cultural evolution identifies areas for improvement and ongoing challenges, essential for measuring strategic impact. Monitoring changes enables organisations to regulate their approaches based on empirical evidence, ensuring efforts remain relevant and impactful. Additionally, assessing change fosters accountability, as progress can be objectively measured and communicated to stakeholders [69].

(g) Detailed breakdown

Quantitative surveys reveal cultural differences across departments by disaggregating data according to demographic and organisational variables. The observed outcomes help to identify areas with advantages and limitations, guiding targeted interventions. For example, should a department receive a low rating for food safety practices, tailored training can address these challenges. This systematic strategy ensures nuanced and actionable cultural assessments, addressing different departments’ specific requirements within the organisation [39].

(h) Foundation for follow-up

Quantitative surveys establish a foundation for follow-up qualitative studies. The structured survey data highlights areas for further investigation. For example, low scores on FSC aspects such as employee training require further investigation through interviews or focus groups, facilitating deeper insights into the identified concerns. As a result, this suggests a sequential integration of first quantitative data collection, informing the subsequent qualitative method, resulting in a broad scope and analytical depth in cultural assessments. Therefore, qualitative follow-up investigation provides context to quantitative findings, uncovering foundational drivers and providing actionable insights informed by employees’ experiences [66].

(i) Content development and mapping

The element of compliance introduces an additional challenge to the scientific context of assessments. In this case, articulation with the standard requirements is necessary to guide the interventions amidst differing nomenclatures to meet the certification requirements. For example, some assessment instruments may indicate wording such as “inspiration”, whereas the standard may require evidence of an “inspired” food handler workforce. A further complexity arises from the multitude of assessment categories and questions, which are often categorised under specific constructs, but may overlap conceptually, drawing on aspects that may manifest across multiple qualitative response areas.

**Figure 3:**
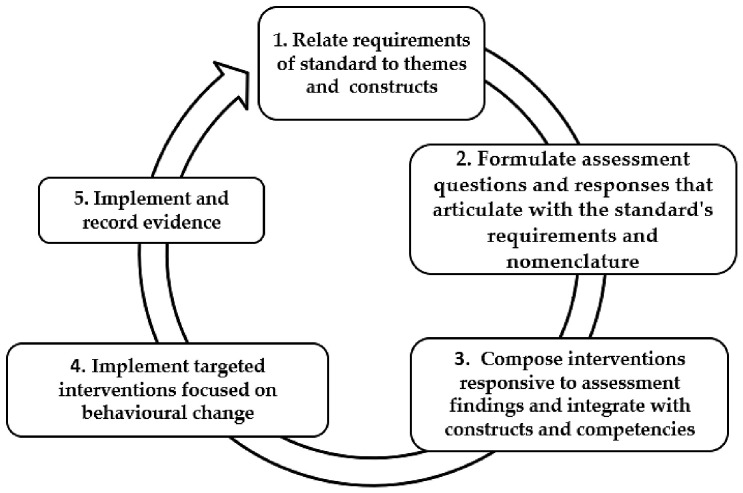
The alignment of constructs, standards, and interventions.

Figure 3 illustrates the alignment of these elements and constructs with standard requirements, presenting the overlaps and interplays. Leadership serves as a further example of a multidimensional construct, encompassing various interrelated sub-constructs, such as leadership development, and including matters such as communication, empathy, trust, integrity, and the like. The Food Safety System Certification 22000 (FSSC 22000) guidance document, published in July 2023, incorporates updates that align with Version 6 of the FSSC 22000 Scheme, and the Global Food Safety Initiative (GFSI) benchmarking document outlines the requirements that must be implemented by GFSI-approved Certification Program Owners (CPOs) and audited onsite [4]. In alignment with clause 5.1 of the International Organisation for Standardisation (ISO) 22000:2018, organisations committed to fostering a positive food safety and quality culture are mandated by top management to establish, implement, and maintain specific food safety and quality culture objectives as integral components of the management system [4].

The ISO 22000:2018 standard underscores the significance of elements such as food safety communication, training, employee feedback and engagement, and performance assessments, thereby underscoring the essential role of qualitative methods. Table 4 provides a summary of the additional requirements concerning food safety and quality culture, as included in FSSC 22000 Version 6, under Part 2 of the Scheme. The themes identified in the standard emphasise leadership, roles and responsibilities, training, and decision-making, with interviews frequently cited as a pivotal tool for measurement [4].

The integration of these elements highlights the critical role of qualitative data collection methods in enhancing an organisation’s FSC. Therefore, reflecting a broader recognition of the need to understand and cultivate the underlying human factors that contribute to effective FSMS. The current research paper focuses on recognising a variety of resources and behavioural factors essential for fostering a robust FSC. As identified by Frankish et al. [77], these resources include time, financial investment, infrastructure, equipment, research, staffing, effective communication, training, access to information, regulatory support, technology, and clarity in guidelines. The behavioural factors that play a vital role in shaping a robust FSC may consist of leadership, awareness, individual accountability, teamwork, capability, understanding, commitment, motivation, self-belief, and effective supervision. These elements of resources and behavioural factors collectively form a holistic framework that is pivotal for enhancing the efficacy and efficiency of safety initiatives in the food manufacturing sector.

(j) Formulating constructs and questions

Due to increasing survey fatigue, with resulting challenges in response rates, it is important when formulating constructs and questions to ensure that the intended outcome is attained with the least inputs and time required by ensuring a clear, logical, and streamlined process. Peripheral to the mainstream questions responding to the overarching theme are those considerations that are used via general good practice in terms of structured questionnaires. Although the overarching theme of FSC, as applied and assessed in the standard, serves as the primary framework, it remains essential to adhere to established methodology in developing data collection tools, in particular structured questionnaires.

At the core of quantitative and qualitative assessments lies the existence of clear and unambiguous questions/themes that need to be formulated to ultimately retrieve the information required. However, a notable challenge in thematic construction lies in the diverse interpretations of questions, which are often influenced by the semantics, language, and vernacular inherent to various industries, regions, and cultures [78].

**Table 4:** The FSSC 22000 guidance document and associated interventions [4].

Elements	Themes	Qualitative Methods	Role(s)	ISO 22000:2018
Communication	Leadership, commitment	Observation	All employees	Clause 5.1
	Food safety policy	Observation	All employees	Clause 5.2.2
	Vision and mission	Observation	Top management	Clause 5.2.2
	Roles and responsibilities	Interview/ observation	Shop floor	Clause 5.3.1
	Communication system	Interview/ observation	Shop floor	Clause 7.4.3
	Food safety expectations, decision making	Interview	Top management, shop floor	Clause 4.2, Clause 6.2
	Response time	Observation	Top management	Clause 4.1, Clause 6.3, Clause 9.3
	Industry intelligence	Interview	Top management	Clause 4.1
Training	Food safety training frequency	Interview	Shop floor	Clause 7.2
	Individual contribution	Observation	FSMSs	Clause 7.3
Employee feedback and engagement	Responsibility to report FSMS problems	Interview	Food safety leader, shop floor	Clause 5.3.3
	Individual contribution	Interview	Shop floor, observation	Clause 7.3, c
	Individual commitment	Interview	Shop floor, observation	Clause 7.1.2
	Documentation design	Observation	FSMSs	Clause 7.5.1
	Engagement	Observation	Food safety team, top management	Clause 5.3.2
Performance measurement	Measurement	Interview/ observation	Shop floor	Clause 9.1.2, Clause 9.3.2
	Change management	Interview	Top management	Clause 6.1.1
	Complaints and non-conformances	Observation	Top management	Clause 9.1.2, Clause 10.2
	Changes and improvement to FSMSs	Observation	Top management	Clause 8.9.1, Clause 10.3

Table 5 presents a summary of methods to guide best practices in the formulation of assessment questions, whereas Table 6 provides a generic template that comprises constructs derived from various thought leaders [13,21,69,79] and is adapted for specific scenarios. The prototype questions may be adapted/used as a quantitative survey data collection instrument to use as a general FSC assessment.

Currently, the standard/s accommodate the industry by allowing compilations of its own assessment instruments, which allow particular companies to enhance data collection tools to align with their specific industry and the traditions, languages, and vernacular of specific environments and regions. The last section of the questionnaire (Table 6), “Intervention preferences”, provides a pre-emptive advantage in that it supports the alignment and intervention strategies by offering insights into the types of interventions (not themes or constructs, but enablers and platforms) that are deemed effective and acceptable by respondents.

#### 4.2.3. Qualitative Data Collection

To effectively assess and enhance FSC within an organisation, it is essential to employ qualitative data analysis that focuses on the intricate dynamics between leadership and culture. Qualitative methods provide comprehensive insights into FSC and leadership, leading to more effective interventions and a stronger commitment to food safety. These methods also have limitations, including being time-consuming, managing substantial amounts of data, and requiring rigorous analytical techniques, such as thematic analysis. Moreover, qualitative methodologies, including focus groups and interviews, primarily rely on self-reported data, which may be subject to various biases [68].

Notwithstanding its robustness, qualitative data is critical in identifying contextual factors and variations that influence FSC. Unlike quantitative methods, qualitative methods such as interviews, focus groups, and observations investigate the intricacies of employee attitudes and behaviours, providing a holistic view of FSC. Johnson et al. [34] highlighted that qualitative data can provide a comprehensive assessment of FSC by revealing the underlying motivations and perceptions that drive food safety practices. Comprehending these differences is vital for designing targeted interventions that address the root causes of food safety concerns. Utilising qualitative assessment methods ensures that the assessment of FSC is comprehensive and reflects the organisation’s current state. Incorporating diverse perspectives promotes a collaborative approach to food safety, cultivating a culture in which all contributions are valued and leveraged to enhance food safety practices [80].

Root cause analysis in food safety, facilitated by qualitative data analysis, uncovers underlying factors contributing to shortcomings in food safety. According to Nielsen [81], qualitative methods can identify specific concerns affecting a safety culture within organisations, such as poor training and communication. Addressing these root causes enables organisations to develop targeted interventions to resolve existing concerns and to prevent future problems, thereby strengthening the overall FSC. Gualandris et al. [82] further argued that the use of qualitative indicators could enhance health and safety practices within supply chains. Continuous feedback derived from qualitative assessments allows organisations to regularly evaluate and refine their food safety practices, thereby promoting a culture of continuous improvement and enhancing FSC.

(a) Focus groups, interviews, and qualitative comments in surveys

These data collection approaches accommodate the elicitation of responses and are well-established qualitative research methods employed to gather detailed information and insights from respondents, each offering unique benefits and characteristics to support various objectives. Focus groups are particularly advantageous for exploring a broad spectrum of opinions, generating ideas through group interaction, and observing group dynamics. The interactive nature of focus groups encourages participants to engage in discussions, often resulting in deeper insights [83]. The inclusion of multiple participants facilitates the collection of a range of perspectives, thus assisting in identifying common trends and diverse opinions within a target group [84]. Additionally, focus groups are time-efficient and cost-effective as they enable assessors to collect data from several participants simultaneously [85]. The observational opportunities provided by focus groups allow interviewers to capture group dynamics, body language, and reactions, and, in such a way, add context to verbal responses [86]. However, focus groups have certain limitations. Participants may be influenced by dominant voices within the group, potentially resulting in conformity or suppression of dissenting opinions [87]. Focus groups may also cause additional challenges in terms of anonymity, in that participants may not always trust each other not to divulge responses. While focus groups provide a breadth of information, the depth of individual insights may be less than what can be obtained through interviews [85]. Coordinating schedules for multiple participants can be challenging, and the setting must be conducive to group discussions [83].

In contrast, interviews offer the advantage of producing in-depth data through the exploration of individual experiences, thoughts, and feelings, often providing richer and more detailed insights [88]. The one-on-one nature of interviews can encourage participants to share more openly and honestly, especially on sensitive topics [89]. Interviews also provide flexibility, as these can be tailored to the individual, enabling deeper probing based on the participant’s responses [90]. Without the presence of others, participants are less likely to be influenced by peer pressure, thereby promoting more genuine responses [91]. However, conducting individual interviews is generally more time-consuming and expensive compared with focus groups [92]. Since interviews are conducted one-on-one, the facilitator gains insights from fewer people, which might limit the diversity of perspectives [90]. Additionally, the presence and behaviour of the interviewer can significantly influence the responses, potentially introducing bias [93].

Similar to the importance of effectively designing survey themes, the design of agenda items to prompt participant feedback and participation during focus groups, as well as to ensure a safe and structured environment for engagement, is pivotal. Table 7 presents a generic template for an FSC focus group assessment, including advice and best practice indicators.

(b) Observational assessments

Observational assessments are often employed to enhance the robustness of qualitative information. Observational assessments allow the collection of real-time data on participants’ behaviours and interactions, providing an independent measure to verify self-reported information in the food service environment, as a triangulation method. Triangulation is a fundamental strategy in qualitative research that enhances the credibility and validity of findings by using multiple data sources or methods [94]. One benefit of observational assessments is their ability to capture non-verbal cues that are often overlooked or underreported in verbal communication [95]. Non-verbal behaviours, such as facial expressions, gestures, body language, and social interactions, can provide deeper insights into participants’ true emotions, attitudes, and social dynamics. These non-verbal behaviours enrich the data and offer a better understanding of the participants’ perspectives. Zanin et al. [18] conducted a longitudinal study on a food service case and documented the proactive evolution of FSC through detailed observations of daily routines.

Facility observational assessments and walk-throughs are helpful tools for evaluating and enhancing FSC within a food production or service environment. These assessments involve observing food safety awareness messaging and signage, practices, and behaviours as these occur in real-time within the facility. Observational assessments provide further insights into inter-collegial and line function dynamics, safety, and hygiene protocols, the state of the environment and equipment. Observational assessments are often underestimated as data collection instruments because of the ability to discern contextual elements, especially considering that the observer is experienced in the field. Ultimately, observations help gauge the level of employee commitment to food safety and, most importantly, the level of leadership engagement and trust. Additionally, regular observational assessments have the further benefit of providing a strategy for continuous awareness beyond the formal FSC assessments and interventions, thereby responding to the requirement of continuous improvement [96].

The combination of observational assessments with focus groups and interviews facilitates a deeper level of analysis. Observational data can reveal patterns and behaviours that participants may not consciously recognise or articulate during focus groups and interviews [97]. This depth of analysis allows FSC assessors to identify emerging themes and variables that enrich the overall understanding of the FSC topic. For example, in organisational studies, observing workplace interactions can uncover underlying power dynamics, informal communication networks, and cultural norms that influence organisational behaviour, providing a richer analysis than that derived solely from focus groups or interviews [97]. Table 8 provides a generic walk-through checklist to formalise facility observations and walk-throughs.

#### 4.2.4. Quantitative Analysis and Performance Indicators

Representation of results by means of descriptive statistics is generally adequate in the majority of FSC assessments, with inferential statistics (primarily correlation and significant differences) used optionally depending on the depth of the analytical demand. Statistical techniques may also express a general response to determine the general maturity score and identify areas of strength and improvement. The calculations are usually performed manually or processed with the use of statistical software, such as SPSS (version 30). Regarding maturity calculation, the methodology described in the table below (Table 9) is proposed, with 80% selected as an acceptable performance benchmark. It is important to note that the maturity model serves as a comparative tool, rather than a benchmarking tool. Namely, it is used to compare sub-strata of a company, such as departments or regions, rather than calculating conformance to a particular limit. The reason for this is that it is unlikely, because of the diversity of industry cultural assessment methods, to indicate a performance measure. Comparing the FSC scores of sections and departments provides an effective tool for enabling competition during future surveys, as required by the standard’s continuous improvement requirement.

Should a benchmark be set internally, various approaches may be considered in selecting, for example, an 80% score as being compliant. These approaches include consideration of vital minority effects, industry practicality, using balanced and holistic approaches, and keeping in mind the contextual interpretation. By delving deeper into quantitative data, clusters can be “harvested” in order to obtain results pertaining to a specific section in order to contextualise an identified risk or outcome. By concentrating on a vital subcategory, targeted solutions can be proposed and compared with others that may be overarching [98]. This principle directly informs the use of the 80% maturity score as a performance benchmark, aligning it with general human resource performance standards. Similarly, in Likert scale interpretations used to assess organisational culture, an 80% benchmark is often applied, promoting continuous improvement while acknowledging areas for growth [99]. By leveraging the 80% rule in both performance and cultural assessments, organisations can effectively drive performance and foster an environment of sustained excellence and strategic focus.

In FSC assessments and surveys, scoring 80% or above may indicate a strong cultural foundation, where the majority of employees feel engaged, motivated, and aligned with the organisation’s values and goals. It serves as a benchmark suggesting that the most essential aspects of culture are well-established and positively influencing organisational performance. While perfection (i.e., 100%) may be ideal, it is often unrealistic across all cultural dimensions. Aiming for an 80% score acknowledges the challenges of attaining complete alignment, but still reflects a high level of satisfaction and effectiveness without imposing unattainable standards. The precise score may vary depending on the organisation, industry norms, and specific cultural goals.

(a) Descriptive and Inferential Statistics

**Figure 4:**
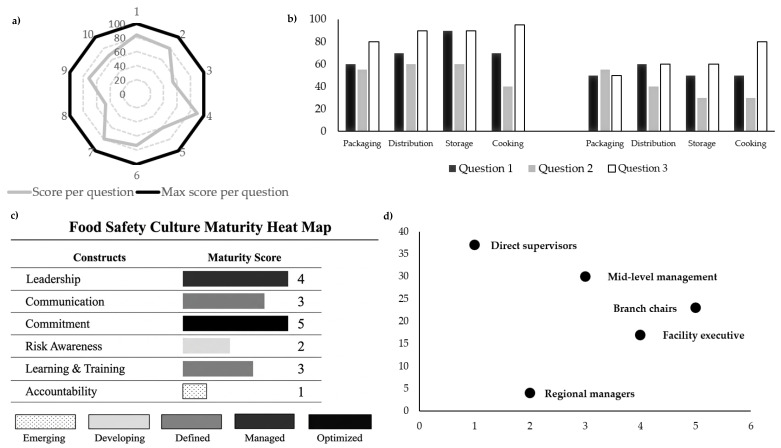
Visualisation of FSC data to enable comparisons, trends, outliers, and magnitudes: (**a**) a change in appetite trend; (**b**) constructs showing vision and empowerment at various sections and post levels; (**c**) a visualised maturity heat map; and (**d**) staff perceptions of leadership commitment.

Considering the steps listed under the verb level in column 3 of Table 9, the first and most pivotal is “visualise”, as this enables the reader to grasp the findings in a graphic manner, identifying trends, outliers, tendencies, comparisons, and the like (Figure 4). Prior to visualisation, the capturing and coding of collected data should be performed, either manually or electronically. Quantitative coding assigns numeric values to closed-ended responses (e.g., a 1–5 scale), allowing for statistical analysis and easy comparison. Contemporary methods utilise software-assisted coding to identify recurring themes related to cultural aspects, whereas data capture is automated in the case of online or electronic surveys [100]. Table 10 provides a summary of popular and effective methodologies and tools used to visualise collected FSC data using various software programs.

Inferential perspectives extend the interpretation process by enabling assessors to organise, summarise, elaborate, comprehend, and evaluate data, as further depicted in Table 10. These methods facilitate the drawing of conclusions about the data, using techniques such as hypothesis testing, confidence intervals, regression, and correlation analysis [68].

(b) Competency in FSC Analysis

To ensure that the correct findings and recommendations are achieved in the assessment process, the necessary competencies are required by either external consultants or the internal delegated departments responsible for the FSC assessment. The development of analytical skills necessary for critically evaluating information, identifying patterns, and making informed decisions may be an effective strategy for ensuring continuous improvement. FSC assessors must be able to apply analytical thinking to interpret assessment findings, evaluate the validity of conclusions, and identify potential limitations or biases in FSC assessment designs.

Critical thinking is a core component of analytical skills that enables assessors, whether internal or external, to question assumptions, consider alternative interpretations, and draw logical conclusions [101,102]. For example, in the field of environmental science, researchers employ various analytical techniques to assess the impact of human activities on ecosystems and develop strategies for sustainable resource management [103]. Through refining analytical skills, the FSC assessment intervention can become a capacity-building exercise in itself, enabling designated staff to conduct rigorous analysis and produce reliable evidence to inform policy decisions and conservation strategies.

As part of analytical competency, cognitive progression entails the ability to adapt to new information, technologies, and methodologies. The pace of scientific advancement that forms and enhances cultural investigations is rapid, and FSC assessors, whether internal or external, must continuously update their knowledge and skills to remain relevant and to adapt to continuous improvement requirements with demands beyond the first round of status quo assessments. This adaptability is particularly crucial where changing standards and compliance requirements, emerging trends, technologies, and societal needs constantly reshape risks and priorities. For example, advancements in digital technologies have transformed data collection, analysis, and dissemination across fields, such as food production, healthcare, education, and environmental science [104]. Food companies that embrace these technologies elevate their agility and responsiveness, and ultimately are better positioned to produce timely and impactful outcomes that address contemporary challenges.

**Table 9:** Options for visualising quantitative FSC data analysis results.

Graph/Table	Purposes	Applications	Motivations
Bar chart	Comparing quantities across categories and departments.	Categorical data comparisons (e.g., survey results and sales figures).	Bar charts clearly compare data across categories, making it easy to see differences in size or frequency. They are simple to understand and versatile, especially when providing feedback to food handlers.
Histogram	Showing distribution of continuous data.	Understanding distribution, central tendency, and spread of data.	Histograms help identify patterns, such as skewness, normality, and variability, in continuous data, aiding in statistical analysis.
Pie chart	Representing proportions of the whole facility.	Showing percentage breakdown of categorical data.	Pie charts are effective for visualising proportions, making it easier to grasp the relative importance of parts within a whole.
Line graph	Displaying trends over time.	Time-series data (e.g., sales over months or website traffic).	Line graphs are ideal for illustrating trends and changes over time, highlighting patterns or seasonality in data. FSC continuous improvement is clearly presented in line graphs.
Scatter plot	Showing relationships between two variables (e.g., products).	Correlation analysis (e.g., salary vs. performance or post level).	Scatter plots help visualise relationships, correlations, or clusters between two continuous variables, providing insight into patterns and outliers.
Box and Whisker plot	Visualising distribution, medians, and outliers.	Comparing distributions of datasets and detecting outliers.	Box plots are effective for summarising data distribution and spotting outliers, especially when comparing multiple groups.
Stacked Bar	Displaying composition of categories within groups.	Comparing subcategory contributions across different groups.	Stacked bar charts allow for comparing overall sizes and the composition of categories, showing how different components contribute to a total.
Heatmap	Visualising patterns in data via colour intensity.	Correlation matrices, survey ratings, or performance data.	Heatmaps are useful for identifying patterns or correlations in large datasets, providing a quick visual cue through colour intensity. This is especially valuable in visualising food hazards and risk categories.
Correlation matrix	Showing relationships between multiple variables.	Analysing correlations among multiple variables.	Correlation matrices quantify relationships between variables, helping to identify which factors are most closely related. Valuable in improvement.
Summary Table	Presenting descriptive statistics.	Overview of statistics (e.g., mean, median, and standard deviation).	Tables of summary statistics provide a quick snapshot of data properties, aiding in data interpretation and comparison.

**Table 10:** Selected inferential statistics options to inform FSC relationships, facilitate decision making, and determine probabilities (e.g., inter-branch, inter-facility, inter-department, supervisor levels, commodities, etc).

Features	T-Tests	Correlation Analyses
Purpose	Compares the means of two groups to determine if they are significantly different from each other.	Measures the strength and direction of the relationship between two continuous variables.
Data type	Used with categorical data (groups) and continuous data (e.g., scores).	Used with continuous data (e.g., satisfaction scores, and performance metrics).
Example	Comparing employee satisfaction between two departments (e.g., HR vs. IT).	Investigating the relationship between employee satisfaction and perceived leadership quality.
Use case	To assess differences between two independent groups.	To identify and quantify relationships between two variables.
Hypothesis tested	Null hypothesis: no difference between group means. Alternative hypothesis: there is a significant difference.	Null hypothesis: no relationship between the variables. Alternative hypothesis: a significant relationship exists.
Outcome	A *p*-value to determine if the mean difference between groups is statistically significant.	A correlation coefficient (e.g., Pearson’s r) indicating the strength and direction of the relationship.
Assumptions	Data should be normally distributed, groups should be independent, and variances should be equal.	Assumes linear relationship between variables and normal distribution for each variable.
Result interpretation	A significant *p*-value indicates a meaningful difference between groups.	A correlation coefficient close to +1 or −1 indicates a strong relationship, while 0 indicates no relationship.
Type of data comparison	Compares means between two groups.	Examines the degree of association between two variables.
Example in organisational culture	Comparing job satisfaction scores between employees in different departments to see if one department has a significantly higher score.	Exploring whether there is a relationship between employee satisfaction and the perception of management support.

A T-test is used to compare two groups (e.g., two departments or two time points) to see if their means differ significantly. It is best suited for testing differences among groups. Correlation analysis is used to explore the relationship between two continuous variables (e.g., satisfaction and motivation), helping to quantify how closely the two variables are related.

Apart from generating results that inform FSC, a fundamental and likely most challenging competency is to suggest implementable solutions through the generation of novel ideas, approaches, and answers that diverge from conventional thinking [105]. Creativity and the confidence to implement FSC improvement strategies, beyond only training, require creativity and innovation - traits that facilitate original interventions, methodologies, or technologies that address FSC improvement needs or challenges. For example, food engineers frequently rely on creative thinking to design new technologies or improve existing ones [106]. Ultimately, whoever is assigned to implement effective and responsive FSC initiatives should boast competencies in engaging diverse perspectives and interdisciplinary considerations throughout the food safety and production portfolio of a particular facility, collaborating with line management and experts from sections and departments, and being in touch with emerging technologies and inventions. Table 11 presents a progression model to guide cognitive arguments to translate data into creative solutions.

#### 4.2.5. Presenting the Bottom-Line

Therefore, the presentation of the findings and the consequences and interpretations thereof have to be funnelled into understandable and clear-cut outcomes, understandable across all levels of the facility. Presenting FSC assessment findings in a simple, at-a-glance format further enables management to clearly understand issues and areas for improvement without being overwhelmed by complex data. Clear, concise findings assist line management, the quality and safety team and, ultimately, executives to prioritise actions and allocate resources efficiently. This clarity also enhances communication within teams, ensuring faster and more effective improvements in food safety practices.

FSC maturity models provide an effective means of enabling the interpretation of results “at-a-glance”. Such models enable clear benchmarking, identification of gaps, progress tracking, and the development of improvement plans, as well as supporting strategy alignment. The maturity model presented below (Table 12) provides a methodology to enable the clear and easy interpretation of FSC assessment information [62]. The maturity model provides a framework for assessing the level of an organisation’s food safety practices and culture, ranging from basic to advanced stages. The model also assists with identifying strengths and areas for improvement by evaluating factors such as employee awareness, leadership commitment, and adherence to safety protocols. Therefore, the model provides a structured path for food production facilities to progress toward a stronger, more proactive FSC over time.

In terms of calculations, Table 13 proposes several options, either by calculating average scores across questions, constructs, departments, etc., and funnelling such numbers to enable composite scores. In the example provided, evaluation categories are allocated to assign a value or outcome to the particular score that relates to a selected performance measure. These evaluation categories (dimensions) include: (1) sustaining excellence; (2) proactive and preventative; (3) safeguarding; (4) aware and responsive; and (5) reactive and compliant. This approach is also effective in providing a benchmark against which to measure future continuous improvement successes. In summary, the interpretation of the model provided by Lues [62] enables the following:

- Comparison of statuses and progress across dimensions and scales.
- Comparisons of maturation scores across constructs.
- Scoring across sections of the facility, branches or regions—beneficial for enabling comparisons and, if managed effectively, may form a basis for constructive competitions.
- Contrasts over time to support continuous improvement, as measured during consecutive assessments and audits.

Effective FSC maturity comparisons amongst independent industries remain challenging. Because of the vast differences in the farm-to-fork food continuum, spanning not only different commodities and processing dynamics, but also geopolitical and cultural domains, linking quantitative performance indicators (KPIs) to FSC successes remains impractical. Maturation categories are subjective and internally focused, rather than providing a direct comparison to other industries and scenarios. Instead, the categories provide a framework against which the organisation can compare departments, sections, and branches and respond accordingly.

**Table 13:** Food safety culture maturity scoring model.

Constructs	Vision		Inspiration		Empowerment		Performance		Change in Appetite Improvement	
Dimensions and Scales	The existence of clear direction and understanding of food safety goals, expectations, and targets, encouraged by ethical, logical, and compassionate leadership through effective communication and across all occupational levels.		The confidence and participation of employees on all levels to engage in food safety, facilitated through actions that create a sense of loyalty, ownership, responsibility, and confidence.		The provision of and access to adequate and relevant training and resource provision, including knowledge and skills, infrastructure, equipment, finances, and staff to enable achievement of the organisation’s food safety goals and expectations.		Maintenance of responsive and consistent documented food safety management systems and procedures which includes relevant compliance and risk assessment instruments, monitoring, metrics, evaluation, consequences, achievements, and improvement measures.		Our organisation’s level of awareness, respect, and equitability across portfolios relating to culture, race, gender, and levels of authority; its agility and progressiveness to change and improvement in response to emerging technological, economical, societal, and organisational demands that may influence food safety performance.	
	QN	QL	QN	QL	QN	QL	QN	QL	QN	QL
Sustaining Excellence	Maturity Level 5 Score 95% above		Maturity Level 5 Score 95% above		Maturity Level 5 Score 95% above		Maturity Level 5 Score 95% above		Maturity Level 5 Score 95% above	
Proactive and Preventative	Maturity Level 4 Score 86–95%		Maturity Level 4 Score 86–95%		Maturity Level 4 Score 86–95%		Maturity Level 4 Score 86–95%		Maturity Level 4 Score 86–95%	
Safeguarding	Maturity Level 3 Score 71–85%		Maturity Level 3 Score 71–85%		Maturity Level 3 Score 71–85%		Maturity Level 3 Score 71–85%		Maturity Level 3 Score 71–85%	
Aware and Responsive	Maturity Level 2 Score 51–70%		Maturity Level 2 Score 51–70%		Maturity Level 2 Score 51–70%		Maturity Level 2 Score 51–70%		Maturity Level 2 Score 51–70%	
Reactive and Compliant	Maturity Level 1 Score up to 50%		Maturity Level 1 Score up to 50%		Maturity Level 1 Score up to 50%		Maturity Level 1 Score up to 50%		Maturity Level 1 Score up to 50%	
QUANTITATIVE MATURITY SCORE (QN): ___ QUALITATIVE MATURITY SCORE (QL) ____ TOTAL MEAN MATURITY:____										

#### 4.2.6. Qualitative Data Analysis and Interpretation

Qualitative information is very helpful in providing reasons and context for quantitative findings and visual observations in food production environments. Qualitative data analysis involves methods for interpreting and making sense of non-numeric data, such as interviews, focus groups, and open-ended survey responses. Data interpretation is, however, preceded by effective capturing. This capturing of qualitative data has historically been a manual/written exercise, supported by verbatim audio recordings [68]. Contemporary technologies facilitate the transcription of audio to written text, thereby providing options to seamlessly conduct further qualitative data analysis. With relevance to FSC, prominent qualitative analysis and interpretation methodologies include:

- Thematic analysis: Identifying, analysing, and reporting patterns or themes within the data. It involves familiarising oneself with the data, coding, generating themes, and interpreting patterns.
- Content analysis: Systematically categorising and interpreting textual or visual data to identify the presence of specific words, themes, or concepts. It can be both qualitative and quantitative.
- Narrative analysis: Examining the structure of stories or personal accounts to understand how individuals make sense of their occupational experiences and the meaning they attach to them.
- Discourse analysis: Analysing language and communication practices to explore how social phenomena, power dynamics, and ideologies are constructed through language.
- Case study analysis: Focusing on in-depth examination of a specific case (individual, group, event, etc.) to gain detailed insights and generate broader understanding.

**Figure 5:**
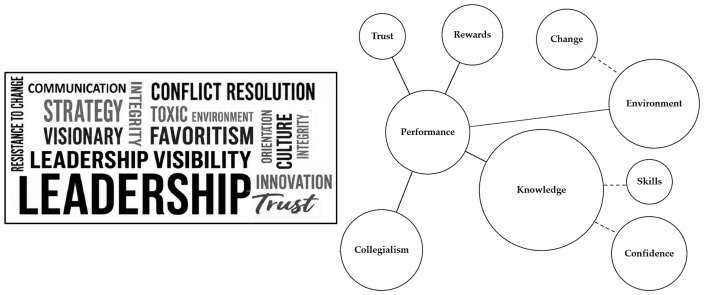
Examples of processing and visualising thematic qualitative responses via word clouds and mind maps (The dashed lines represent secondary associations).

The visualisation of qualitative observations is equally important to initiate the interpretation process (Figure 5). The use of methodologies such as Venn, relationship, process, cycle, hierarchy, Hub and Spoke Designs, models and mind-maps is useful in demonstrating trends and relationships [78]. More recently, word cloud technology has been increasingly used to interpret qualitative data. These are visual representations of qualitative data that highlight the most frequently occurring words or phrases in a dataset. The size of each word corresponds to its frequency, with larger words indicating greater prominence in the data. This method is useful for quickly identifying themes, concepts, or topics within a set of open-ended responses, allowing easy identification of trends and patterns in the qualitative data [108].

### 4.3. Phase 3: Alignment of Gaps and Solutions

The gap analysis and alignment phase of the FSC pipeline (Figure 2) refers to the linking of findings with priority needs and gaps to inform targeted interventions. This phase is pivotal, as it is aimed at aligning the findings with effective intervention strategies. The challenge with this phase is to navigate the process through the multitude of general, established interventions offered by the broader industrial psychology-related interventions. The selection of interventions from the proverbial “shelf” may prove effective to some extent (while it is important to acknowledge the peculiarities and uniquenesses inherent to the food safety narrative) and ensures targeted and effective interventions. As referred to in Table 14, the third phase of the FSC intervention entails creative and solutions-based competencies to enable the extension of the assessment findings into tangible recommendations and solutions that are effective and aligned with the standard requirements. The aim of the previously discussed mapping exercise is to articulate the assessment constructs with the standard requirements, which should significantly support the seamless identification and implementation of solutions and interventions. Although the themes and constructs may be relatively clear, the enablers and types of interventions are likely to require in-depth consideration, as these are informed by a wide array of respondent and organisational characteristics, such as level of education, language, interpersonal dynamics, logistics, and culture.

#### 4.3.1. Resources and Approach

Depending on the size and complexity of the facility, receiving thousands of datasets from qualitative and quantitative assessments, originating from large numbers of respondents, various sections, levels of authority, different languages, and similar variables, can be highly demanding. Synthesising such responses into actionable and appropriately targeted interventions presents a significant challenge. Similar to this, the various constructions, data collection, and corridor discussions, the multitude of possible intervention strategies, and the process of data interpretation and recommendation may create further challenges. Fortunately, the standards currently allow for a phased approach that demonstrates that the company is considering the matter of FSC and is committed to continuous improvement. Therefore, the sample sizes and related interpretation dynamics do not have to be insurmountable. It is also pertinent to consider the resources and approach of the interpretation and recommendations process, in terms of technological and human resources.

#### 4.3.2. Expert Review and Participation

Rather than delegating the function to align assessment findings solely to an external or internal service provider, a panel of peers and experts should be convened to collaborate and engage on the findings, to inform relevant solutions. Such panels should constitute representatives from the assessed food facility, members of the external assessment team, and independent external representatives with a knowledge of the field or industry. When convening such a panel, confidentiality and limited personal interest and bias should be ensured. The aim of the panel should thus be to dissect and engage on the assessment findings and contribute inputs from various contexts. This process should be discreetly and objectively facilitated to ensure objective and informed inputs and limit any possible conflict of interest or commitment. Table 15 summarises the strengths and benefits of utilising peer or expert panels to obtain inputs during alignment of FSC findings with ultimate solutions.

### 4.4. Phase 4: Solutions and Interventions

The manner of approaching and interpreting FSC will ultimately inform the types of interventions to be implemented and is unique to every food production environment. When considering FSC as an overarching concept encompassing all central and peripheral constructs, the intervention scope is likely much broader, with activities relating to compliance included. Alternatively, if FSC is viewed as merely those constructs that influence behaviour separately from compliance, the interventions will be fewer and more targeted [19,30]. Therefore, viewing culture as an overarching concept highlights that culture is not just a force that drives behaviour, but also a product of the behaviours within the organisation. Thus, suggesting a more holistic, interconnected view, where culture includes all elements that drive human actions and is a result of continuous, collective human activity. Conversely, viewing organisational culture as a separate behavioural driver focuses on how culture actively shapes and drives behaviour in a more direct, external way.

Consultants and advisers that view FSMS performance and compliance as an inherent culture component will likely consider interventions related to the basic elements of FSMSs and their peripheral vocational competencies (e.g., competencies in finance, logistics, hygiene, etc.). Fundamentally, this also highlights the arguments about whether FSC should be viewed as a behavioural driver beyond an individual’s primary task description, or whether it should be a monitored and compliant principle only, driven by cause and effect [49]. FSC, as a behavioural driver, places more emphasis on culture as an external force that shapes how individuals act. Conversely, culture as an overarching concept suggests that culture is the context that includes all other forces (such as individual behaviour and organisational norms) that drive actions (refer to Section 3.2). In the former, culture influences individual behaviour, often external to their personal desires, goals, or values. In the collective context, individual behaviours are not just influenced by culture, but are also part of the cultural system, contributing to and defining it. Culture as a driver often suggests a stable, deterministic influence on behaviour, where culture provides the “food safety rules” employees follow. Culture as an overarching concept implies a more fluid, evolving system that is shaped by persistent interactions between individual behaviours, organisational practices, and external influences [60,109].

When considering interventions, it should be noted that if a delegated employee holds functional authority above his/her line of management and is not handled discreetly and respectfully, the scenario can lead to dire consequences for the motivation and dedication of the subordinate. Additionally, the distinctiveness of race and gender, elder respect, religious dispositions and views, cultural influence, tribalism, language barriers, and the like support the notion that cultural interventions and approaches should be well thought-through [53]. Similarly, occupational levels, divisions, departments, sections, regions, factories, seasonal versus permanent versus contract workers, education levels, etc., add to the plethora of considerations when composing interventions.

Whether the organisation opts to implement the improvements and interventions resulting from the assessment internally, managed by e.g., its own quality assurance or HR sections, or whether the organisation opts to outsource services remains a contested matter. The choice is influenced by considerations such as capacity, resource availability, environmental and societal uniqueness, and the complexity of the required interventions. From a sustainability perspective, the ideal would be to empower the organisation to handle its own continuous improvement initiatives [110]. The services offered by third-party consultants should thus encompass activities aimed at capacitation (i.e., train-the-trainer), rather than retaining clientele through protecting IP and non-disclosure. It is likely that, depending on the circumstances and the organisational dynamics and fluidity, the interventions will constitute a hybrid model with selected interventions managed by outsourced parties, and others remaining in-house.

#### 4.4.1. Enablers (The “How”)

When considering an FSC improvement intervention, the following two basic elements should be considered: (1) the content or themes of the intervention, in other words, the “what”, and (2) the method that will be used to transfer such content, in other words, the “how”. In terms of FSC interventions, overarching themes may be defined as listed below, although these depend on the specific assessment’s findings related to the context and the specific sampling area. These overarching themes should also be dissected into targeted and specific topics. Therefore, the following summarises and presents overarching FSC themes to further inform content development [1,19,25]. Values: The core principles and ideals that guide the organisation’s actions and decision-making processes. These are often articulated in mission statements or value propositions.

- Standards and processes: Rules, expectations, and opportunities that dictate appropriate behaviour within the organisation. These can include dress codes, communication styles, and work habits. It also includes the formal organisational structures, policies, and procedures that influence how work is conducted and how delegations and authorities are distributed.
- Artefacts and symbols: Tangible and visible elements of culture, such as office layout, dress code, company logos, rituals, and ceremonies. These provide a physical representation of the culture. Objects, logos, slogans, and other symbols that represent the organisation’s culture and values. Additionally, these serve as a shorthand for complex cultural norms and values.
- Campaigns: Regularly occurring activities or events that reinforce the organisational culture, such as meetings, award ceremonies, team-building activities, and social get-togethers.
- Reputation and history: Narratives about the organisation’s history, its heroes, and significant events that convey cultural values and provide a sense of identity and continuity.
- Language and communication: The specific terminology, acronyms, and vernacular used within the organisation that reflect its culture and can both include and exclude individuals based on their familiarity with these terms.
- Leadership and management: The behaviours and attitudes of leaders and managers that influence the overall culture. Leadership sets the tone for what is valued and how people are expected to behave.
- Societal cultures and beliefs: Underlying beliefs and assumptions about how the world works and what is important, often taken for granted but deeply influencing behaviours and decision-making.
- Behaviours and examples: The day-to-day activities and behaviours of individuals within the organisation, which collectively shape and reflect the organisational culture.
- Team dynamics and relationships: The nature of interpersonal relationships, teamwork, and collaboration within the organisation. This includes how conflict is managed and how support and cooperation are fostered.
- Resources and environment: These include the tools, equipment, skills, knowledge, vocational knowledge, infrastructure, PPE, mechanisms, technologies, etc., that ensure effective performance, confidence and appreciation.

#### 4.4.2. Themes (The “What”)

To improve the FSC assessment constructs of vision, inspiration, empowerment, performance, and change in appetite and improvement, the following intervention themes may be applied. These themes aim to ensure that the food safety goals, expectations, and targets are clearly understood, communicated, and embraced across all levels of the company [26,33,49].

(a) Vision
  - Committed, Ethical, and Involved Leadership: Ensure leaders at all levels actively instil ethical behaviour, integrate food safety into the strategic mission, and demonstrate visible commitment to inspire organisational alignment. This is relevant not only for the executive, but across the leadership portfolio including first-level supervisors.
  - Clear and Transparent Communication: Establish simple, accessible communication channels (both formal and informal) to share goals, build trust, and utilise cultural storytelling to make the safety narrative relatable. These may include visual signage and displays of food safety values in various departments.
  - Employee Engagement: Cultivate a shared sense of purpose by involving employees in goal setting, establishing clear accountability, and creating open feedback mechanisms for continuous improvement.
  - Vision-Driven Systems and Competence: Align standard operating procedures (SOPs), technologies, and training programs with long-term safety goals, focusing on fundamentals rather than compliance only.
  - Recognition and Collegialism: Foster teamwork across all departments while incentivising and rewarding individuals or teams who actively support the food safety vision.
(b) Inspiration
  - Senior and Peer Role Models: Utilise leaders as active food safety champions and establish peer mentoring systems to build employee confidence and normalise safe practices.
  - Ownership and Inclusivity: Empower staff by assigning specific food safety responsibilities and inviting input on policies, which builds a strong sense of pride and collective engagement.
  - Transparent Dialogue: Maintain a supportive environment where employees feel safe and confident to voice concerns or report hazards openly without fear of retaliation.
  - Gamified Learning and Role Play: Enhance participation through innovative thinking, game-based training challenges and role-play, and by linking food safety mastery to personal development and career advancement opportunities.
  - Feedback and Celebration: Track and display metrics via transparent dashboards, and routinely celebrate milestones or audit successes to maintain high morale.
(c) Empowerment
  - Comprehensive Training and Skills Development: Ensure accessible, role-specific training programs reinforced by practical simulations and hands-on exercises to build real-world confidence.
  - Resource and Operational Capacity: Equip teams with effective and safe equipment, appropriate tools, updated technologies, protective gear, and balanced workloads to ensure safety protocols can be executed without pressure or lapses.
  - Knowledge Sharing and Engagement: Cultivate a cross-departmental learning culture and ensure all employees have easy, on-demand access to internal or external subject-matter and reports.
  - Clear Role Structures and Responsibilities: Provide explicit descriptions of individual food safety roles, supported via delegated authority to respond to risks and raise concerns timeously.
  - Incentives and Rewards: Recognise proactive behaviour through formal rewards. These may include monetary or other means, such as visual displays of “food safety champion of the month”.
(d) Performance
  - Data-Informed Monitoring and Audits: Combine real-time monitoring systems with regular internal audits to track compliance, establish clear benchmarks, and use metrics to drive proactive decisions.
  - SOPs and Documentation: Maintain a robust, easily accessible record-keeping system backed by clear standard operating procedures (SOPs), so that expectations and consequences are understood and monitored.
  - Corrective Actions: Respond to food safety breaches by investigating underlying systemic issues rather than just symptoms, quickly implementing preventive measures to avoid recurrence.
  - Employee Awareness and Knowledge: Train staff specifically on food risks and how to accurately track and document metrics, engaging them directly in the review of performance data to foster ownership.
  - Supply Chain and Consumer Transparency: Extend strict safety evaluations to external suppliers and vendors, while openly sharing performance metrics with customers and regulatory bodies.
(e) Change in Appetite and Improvement
  - Cultural Awareness: Promote diversity in decision-making and educate employees on cultural dynamics to ensure safety initiatives are equitable, fair, and free of bias.
  - Technological Advancement: Proactively evaluate and adopt new tools, digital monitoring software, and equipment to ensure food safety management systems (FSMSs) evolve alongside industry trends.
  - Continuous Learning and Upskilling: Build a growth mindset by offering ongoing training that prepares the workforce to successfully navigate regulatory, environmental, and societal shifts.
  - Cross-Functional Collaboration: Break down operational silos and encourage inter-departmental engagement and accountability.
  - Change Management and Benchmarking: Provide formal change management training to reduce internal resistance to new protocols, while continuously evaluating performance gaps against external industry benchmarks.
  - Wellness initiatives: Establish platforms for feedback and engagement related to aspects such as equity, race, and gender sensitivity, conducive occupational environments, and respect.

#### 4.4.3. Roll-Out and Logistics

This phase of the process entails the actual roll-out and realisation of the interventions identified as responding to identified gaps. Since the identification of interventions aligned with findings has been concluded, this process is realised through the management and implementation of activities such as presentations, campaigns, resourcing, skills development and the like, realised through project management principles [26,60]. Components such as material development, events management, subcontracting, and optimisation of existing tools may be affected. The integration of interventions in terms of practicality, availability of staff, shifts, contact sessions, availability of technology, language considerations, and the like need to be considered. Risk and priority, as well as auditing and assessment urgency, may inform prioritisation and phasing-in. The implementation of the organisational culture improvement strategies requires thoughtful planning and attention to logistical details to ensure smooth execution and long-term success [24,49].

Logistical considerations are crucial for FSC improvement strategies to achieve successful implementation and sustainability of cultural changes. Planning, resource allocation, clear communication, and regular feedback mechanisms are pivotal components for embedding the desired culture into the organisation’s day-to-day operations, especially in large companies. Attention to these logistical factors ensures that cultural improvement initiatives are not only executed smoothly but also lead to continued change [19,96].

(a) Involving support and delegated departments

A successful FSC intervention should commence by engaging and acknowledging the existing resources and departments available in the company to assist and handle. For example, the HR section is likely to boast resources and skills to assist with FSC improvement interventions, whereas the quality assurance and compliance portfolio may handle aspects related to food safety performance, while the resource and infrastructure departments are likely to assist with matters related to environment and infrastructure performance. Aspects such as ownership and resource optimisation may strongly inform the involvement of internal departments, whereas their involvement may complicate matters of confidentiality, exposure, and possible bias. Effective and efficient FSC interventions, regardless of whether managed internally or outsourced, should be guided by a set of well-defined steps and principles, as outlined in the sections below [19,24,60].

(b) Setting the stage

Leadership and organisational buy-in and support are the fundamental requirement to set the stage and expectations for FSC improvement interventions that will follow. Organisational commitment is further needed to effectively translate assessment outcomes into targeted, strategic actions that align with both organisational objectives and relevant standard requirements [24,30]. To maximise impact, assessment results must be tangibly aligned with targeted strategies and standard requirements, backed fully by leadership support. The approach should remain highly flexible, offering either standardised solutions or novel interventions, depending on the unique sector or specific organisational scenario, drawing from general social science principles where appropriate. Structurally, the strategy must incorporate a rich blend of themes and enablers, positioning training as just one component of a much broader portfolio of initiatives. Finally, the process concludes with continuous follow-up feedback and subsequent assessments, embedding these insights into a permanent cycle of continuous improvement to accurately gauge long-term effectiveness.

To ensure strategic and actionable FSC transformation efforts, the following planning elements should be clearly defined and systematically implemented [19,24]. To launch the culture transformation, the company must develop a detailed action plan that clearly outlines the necessary steps, required resources, and designated responsible parties for each phase. This involves setting specific, measurable objectives, such as improved employee engagement and retention rates alongside concrete milestones to effectively track progress. Finally, establishing a realistic timeline ensures that these cultural initiatives seamlessly align with the organisation’s broader fiscal calendars and strategic planning cycles.

(c) Resource allocation

Effective cultural change requires intentional investment [59]. Successful FSC initiatives require the deliberate allocation and assignment of human, financial, and technological resources. This includes dedicating specific team members or establishing a formal culture committee or service providers to oversee ongoing progress. The company should also dedicate a sufficient budget to fund critical activities such as training programs, team-building events, and communication campaigns, while remaining open to investing in internal or external support departments, consultants or specialists.

Leveraging technology to support communication, data tracking, and collaboration is likely to be an effective enabler for FSC improvement strategies [23]. Organisations could utilise employee engagement platforms and HR software to reinforce culture-building activities, use specialised data tools to track critical metrics like satisfaction, retention, and cultural alignment, and optimise digital communication channels like Zoom, Microsoft Teams, or Slack to maintain cultural cohesion in hybrid or remote work settings [23].

(d) Communicating change and progress

Communication is critical in aligning employees and ensuring transparency during FSC improvement and transformation, and a number of practices have been shown to be essential for fostering clarity, consistency, and engagement across the plant or facility [25,111]. To ensure consistent and uniform cultural alignment across all departments, organisations should centralise their communication through platforms like intranets, newsletters, or internal social networks. Crucially, this must be a two-way street; by integrating feedback loops and trusted feedback enablers, companies can share culture-related updates while actively empowering employees to voice their thoughts, concerns, and suggestions.

Supporting individuals and teams through change is vital for long-term success, although this may present a sensitive environment. The following measures offer structured support to navigate the complexities of cultural transition [19,23]:

- Equip managers and leaders with change management knowledge and tools to support their teams through transitions. Additionally, offer workshops or seminars to develop competencies for leading cultural change.
- Establish employee support systems such as coaching, mentoring, or access to HR for guidance during culture transition.
- Provide, through clear and consistent communication, clear guidelines for cultural expectations and how employees can contribute to the changes.

(e) Integration and continuity

For an effective FSC to take root, it must be incorporated into daily organisational activities, and strategies to facilitate the integration of cultural values into daily operations [18,19] should be considered. An effective approach would be to seamlessly integrate their core values into daily workflows, team meetings, and broader operational processes like recruitment, performance reviews, and promotions. This structural alignment is further strengthened by identifying and training internal “cultural ambassadors or legends” who can champion these desired behaviours and advocate for the values directly within their respective teams.

To ensure cultural transformation endures as an integrated part of the organisational identity rather than a one-time project, companies must develop long-term plans to maintain and reinforce cultural improvements over time. Ultimately, these values must be embedded into the organisational DNA, influencing every aspect of operations from core decision-making processes to how success is fundamentally defined and celebrated [25,30].

(f) Training and development

To ensure effective training delivery and accessibility, organisations must plan, schedule, and coordinate highly accessible workshops, seminars, or online courses tailored to accommodate diverse time zones and work schedules [26,53], especially considering the challenging work hours and shifts in many food processing companies. Training programs should be customised to meet the distinct needs of different employee groups, ranging from entry-level staff to middle management and leadership, while aligning with the gaps identified in the FSC assessment [26,53].

(g) Ensuring Consistency

Achieving cultural cohesion across geographically dispersed or functionally diverse teams requires intentional alignment. The following strategies help ensure consistency while allowing for necessary contextual adaptations. For the food sector with its multiple departments, sites, offices, and remote teams, ensuring cultural initiatives are implemented uniformly across all locations and adapting where necessary to local cultures or time zones is pertinent. While maintaining a core set of values, culture-building programs to respect regional differences and unique team dynamics, especially in global organisations, should be aligned. Recognising the diverse backgrounds of employees and ensuring cultural initiatives are inclusive, ensuring representation from different demographic groups, departments, and locations [30].

(h) Celebrating success and acknowledging contributions

Recognising achievements and milestones is a strong enforcer of cultural values and motivates continued engagement. The practices below help embed a culture of appreciation and celebration [1,19]:

- Organise events or recognition activities to celebrate milestones in the cultural change process (e.g., cultural milestones and team successes).
- Communicate the progress and success stories related to cultural improvement in internal newsletters, town halls, or leadership updates.
- Recognise employees who actively contribute to cultural transformation through awards, public acknowledgement, or other forms of recognition.

(i) Tracking, monitoring, and evaluation

To assess the reach and effectiveness of FSC-building efforts, it is essential to monitor employee participation in FSC improvement programs and ensure they are progressing as intended [112,113]. The evidence collected during the tracking will also significantly add to the evidence presented during the auditing process. To foster and track responsiveness, organisations must implement regular surveys and feedback platforms (formal as well as social) to collect feedback on how employees experience cultural shifts [18]. By utilising data analytics to identify trends and gaps, leadership can openly share findings, take corrective action, and maintain an open-door environment where employees feel secure raising concerns or offering feedback without fear of reprisal. To support a data-driven approach to evaluating progress and adapting strategies, organisations must establish clear evaluation mechanisms, such as employee engagement scores, retention rates, and internal surveys, to regularly check the pulse of the company and gauge if cultural initiatives are taking hold. Through leadership reviews, focus groups, and continuous assessment, organisations can ensure these initiatives remain effective and aligned with broader goals, allowing them to remain agile and adjust tactics or re-evaluate the overall strategy if results fall short [1,53].

### 4.5. Phase 5: Internal Developmental Assessment

The principle of continuous improvement constitutes a fundamental component of the food safety culture intent (FSSC 22000 v6). The principle of continuous improvement is more embedded in organisational culture principles when compared with systems, the reason being that cultural improvement is more gradual and more prone to impact from external variables. Although not stated in the standard, the principle of continuous improvement renders the FSC initiative more affordable to the industry, as it allows for a phased approach, either by identifying and addressing priority areas, or by gradually improving the culture through feedback loops. In terms of assessment, this implies that all components of the triangulation toolbox need not be implemented at the outset, but as demanded by deeper and over-time evaluation. If, for example, survey data is deemed adequate to address an array of observations without initially reverting to interviews, focus groups, and facility observations, the latter activities may only be attended to as time advances. Internal, also referred to as developmental, audits play an important role in the effective management of FSC, and are conducted by the organisation itself, or by individuals acting on its behalf. Although ideally, internal audits should only be optional in cases where performance is adequate and compliance guaranteed, such assessments extend beyond, and usually precede, formal third-party compliance audits, and focus on system development and continuous improvement.

Although independent interventions may be considered, FSC developmental audits may be performed internally by the section responsible for safety and quality assurance, or a peripheral section, such as HR, to strengthen independence. As indicated, the competency of the mentioned individuals to conduct the internal audit, focusing on FSC and the standard intent, is vital. While shortfalls in the FSC assessment and intervention process may be observed, these findings are used to drive corrective and preventive actions that build on the FSC improvement findings and solutions identified in the assessment. If such findings necessitate a revisiting of the assessment and intervention process via feedback loops, such a second-round intervention will similarly provide evidence to the upcoming certification audit toward evidence of the organisation’s commitment towards continuous improvement. With reference to Figure 2, the feedback loops (reflection) should ideally articulate between the findings and the specific developmental phase, so as not to repeat actions and activities already implemented as part of the improvement plan. This may require a deeper exploration of the survey findings, consider additional data collection, or revisiting the effectiveness or widening of improvement strategies.

As echoed in the standards, the role of leadership support in the developmental audit phase is important, especially if findings are challenged or may require additional resources. A secondary outcome of the proposed FSC internal/developmental audits is the additional benefit of the strengthening of FSC by providing evidence of leadership commitment, staff awareness, and reinforcing ownership of food safety responsibilities at all levels of the organisation.

### 4.6. Phase 6: Auditing Considerations

Historically, FSMS auditing against standards and requirements has been conducted with a mindset of evaluating systems, records and protocols. The question arises whether developmental and third-party auditors are adequately trained in the narrative of human behaviour, or whether such specialisation is even necessary in the cultural context. A uniform audit approach may not be as effective in the context of FSC, as the considerations and skills contributing to the validity and reliability of audits include, amongst others, the following:

- Audit validity involves ensuring that the audit tools and content utilised in the audit accurately measure the intended constructs and comprehensively address FSC requirements.
- Minimising bias requires maintaining anonymity and confidentiality to encourage honest and accurate responses, while also reducing confirmation and observer bias by limiting factors that may influence auditor subjectivity. Contextual and environmental relevance entails incorporating cultural, societal, and community factors that form part of, or may be excluded from, the audit. This may include adapting the audit to the specific context of the environment to ensure accuracy and relevance.
- Robust data interpretation depends on the use of appropriate analytical and interpretative activities and cognitive progression skills. This may require a broader approach incorporating multidisciplinary and advisory team opinion, triangulation and the like, combining qualitative and quantitative data to provide a comprehensive and accurate understanding of the organisational culture.

The principle that auditing approaches between FSMSs and FSC may vary significantly in terms of focus, methodology, and objectives is demonstrated in Table 16, summarising the differences between the 2 approaches:

## 5. Continuous Improvement

The principle of continuous improvement is clearly indicated as a performance measure in the current standards (e.g., FSSC 22000 v6). The reason for this requirement is, amongst others, to (1) enable industry to navigate the FSC improvement process realistically and within its means; (2) to allow for the development of awareness and knowledge of the FSC narrative amongst countries, societies, and industries; and (3) to ensure continuous progress and positive change in organisations toward excellence. As discussed previously in Section 4, the emphasis on empowerment, rather than reliance on outsourcing, is also significant, as continuous improvement encompasses not only compliance and performance, but also organisational capacitation and long-term sustainability. In addition to the guidance provided by the existing standards to support the continuous improvement process, viz. reporting near-misses, implementing successive interventions, celebrating and acknowledging gains, and updating records. There is a need for deeper consideration of enablers, especially in comprehensive, multi-faceted companies. A constant reflection on and advancement of FSC is essential to ensure consistent adherence to food safety standards, reduce the risk of contamination or violations, streamline food safety processes, proactively identify and mitigate risks, and foster a culture of ongoing evaluation and success. Figure 5 [62] portrays the FSC value chain and contains a visualisation of the prominent reflection steps, particularly between the assessment and alignment, and developmental audit and improvement phases. This framework facilitates revisiting previous steps and considering a re-run based on the effectiveness of the consequent step.

Ultimately, ensuring continuous improvement in FSC is essential for maintaining high standards of food safety, protecting consumer health, and complying with regulations. A strong FSC creates an environment where employees are persistently committed to prioritising food safety in their daily activities, whereas maintaining a culture of continuous improvement in food safety is an ongoing process. Through the engagement of leadership, empowerment of employees, establishment of clear policies, ongoing training of staff, and the strategic use of data and technology, food safety is embedded as a core organisational priority, resulting in enhanced outcomes for consumers, staff, and the company as a whole [30,33]. A continuously improving FSC relies on sustained visible leadership [1,22,30] and a clear, well-communicated vision that encompasses continuous improvement rather than stagnation [25,34]. Operational success requires sustained and transparent communication with supportive reporting mechanisms [34,109], alongside continuous training and professional certification alignment (e.g., HACCP) [60,109]. To sustain and advance this ecosystem, food safety principles must be embedded into daily standard operating procedures (SOPs) [20] and reinforced through a framework of sustained accountability and recognition [23,59].

Progress and improvement should be consistently monitored by combining internal, external, and, especially, internal assessments and self-reflection [23,29] for tracking targeted Key Performance Indicators (KPIs) to eliminate operational stagnation [29,60]. The food sector should increasingly cultivate problem-solving mindsets [19,30] and engage frontline workers in decision-making [22,26], as well as establish robust risk mitigation protocols that are dynamic and responsive to emerging trends [19,96]. This framework is modernised by embracing novel FSMS technologies [20,34] and benchmarking performance against global standards like ISO 22000, BRC, or SQF [49,60]. Ultimately, a culture of continuous improvement means recognising that compliance is not the finish line, and even when standards are met, the maturity of the performance should be progressively raised, pushing the entire organisation to innovate, refine workflows, and adapt to newly emerging food safety risks.

## 6. Contemporary Technologies

The emergence of artificial intelligence, machine learning, AI-driven Natural Language Processing (NLP), predictive analytics, automated qualitative coding, mobile inspection and reporting applications and advanced data visualisation platforms has significantly expanded the horizons of FSC in terms of opportunity and expansion, having multiplied extensively, with such opportunities being both exciting and intimidating [60,100]. These advancements not only enhance the scalability and accuracy of data interpretation and processing but also introduce new considerations regarding ethics and risk. The following section provides an exhibit on emerging technologies with specific application in assessing and advancing FSC.

### 6.1. Fourth Industrial Revolution 4IR

The Fourth Industrial Revolution (4IR) refers to the integration of digital technologies, such as artificial intelligence (AI), robotics, the Internet of Things (IoT), big data, and automation, into various industries. The 4IR is characterised by the blending of physical, digital, and biological worlds, driving innovations and efficiencies that reshape business processes, manufacturing, and consumer experiences [60,100]. In terms of food safety, the 4IR holds benefits for automation and monitoring (AI, IoT sensors, and robotics can monitor food production processes in real time, identifying potential safety issues, such as contamination or temperature deviations [60]. Automated systems can trigger alarms or take corrective actions while reducing human error. Data analytics (big data and machine learning} can analyse vast amounts of food safety data to predict risks, optimise supply chain processes, and identify patterns that may indicate potential foodborne hazards. Blockchain technology enables end-to-end traceability of food products, ensuring transparency and accountability in the food supply chain [114], thereby expediting the identification and resolution of issues, such as contamination or product recalls.

### 6.2. Fifth Industrial Revolution

The Fifth Industrial Revolution (5IR) goes beyond the automation focus of the 4IR and emphasises collaboration between humans and advanced technologies. This articulates strongly with the multi-disciplinary narratives of FSC. The 5IR prioritises the integration of human creativity, empathy, and ethical considerations with AI and machines to address global challenges. The core idea is to ensure technology serves humanity, enhancing well-being, sustainability, and social value [100]. In the context of food safety, the 5IR introduces several transformative narratives. Human and machine interplays contribute to decision-making and oversight and ensure that advanced technologies are deployed ethically and responsibly, with a focus on sustainability [114]. Ethical AI in food safety can be developed to focus on consumer well-being, using data to make informed decisions that prioritise public health and ethical standards, rather than solely maximising efficiency or profit and sustainability and innovation. Moreover, the 5IR emphasises sustainable practices, such as reducing food waste, improving resource management, and ensuring food safety, while minimising environmental impacts. These technologies are applied to create safer, more resilient food systems that align with global sustainability goals.

### 6.3. Lean Six Sigma and Sustainability

The integration of Lean Six Sigma principles with FSC is a strategic approach to enhance both the operational efficiency and the safety of food products, while simultaneously fostering a strong, proactive safety culture [114,115]. When environmental impact is also considered, this integration of FSC and Lean Six Sigma holds significant potential.

- Continuous improvement: Lean Six Sigma’s emphasis on continuous process improvement aligns well with fostering a culture of ongoing learning and enhancement in food safety practices. Both focus on reducing waste (in terms of errors, inefficiencies, or risks) and optimising processes.
- Data-driven decision-making: Lean Six Sigma uses data and statistical analysis to drive improvements, which can help assess and improve food safety practices, identify areas of risk, and make more informed decisions related to safety culture.
- Root cause analysis: The Lean Six Sigma’s DMAIC (Define, Measure, Analyse, Improve, and Control) methodology allows organisations to identify root causes of food safety failures or non-compliance, and create systematic, culture-driven solutions that prevent future issues.
- Employee engagement: Both Lean Six Sigma and a strong FSC require active participation from all levels of employees. Lean Six Sigma encourages team-based problem-solving, which strengthens the involvement of employees in food safety initiatives and enhances their commitment to a culture of safety.
- Standardisation and accountability: Lean Six Sigma helps create standardised, repeatable processes for food safety, ensuring consistency across operations, while a positive FSC reinforces accountability and adherence to these standards.
- Long-term sustainability: The synergy between Lean Six Sigma’s process efficiency and FSC’s behavioural focus leads to sustainable improvements in both operational performance and safety outcomes. Therefore, this integrated approach supports long-term adherence to food safety standards.

### 6.4. Automated Data Collection and Optimisation

This section summarises advanced tools and technologies that enhance the quantitative and qualitative analysis of FSC data, facilitating deeper insights and more effective interpretation of assessment results. Natural Language Processing (NLP) Tools include AI models that analyse open-ended survey responses, interviews, and text-based feedback to identify themes, sentiments, and patterns related to FSC [60,100,114].

Electronic data collection tools, such as Google Forms, SurveyMonkey, and similar platforms, have become integral to modern operational assessments, offering efficient, scalable, and user-friendly means of gathering structured information. These tools enable translation into respondent-specific languages, real-time data entry, automated aggregation, and preliminary analytics, reducing manual errors and improving response rates through accessible online interfaces. In contrast, facility-specific platforms—for example, systems designed and adapted within an organisation’s digital ecosystem, such as Microsoft Power BI—extend these capabilities by integrating data directly from internal sources, dashboards, and performance metrics. Such tailored solutions allow for dynamic visualisation, longitudinal tracking, and contextual analysis aligned with the facility’s unique environment and objectives. Together, these technologies enhance data integrity, facilitate evidence-based decision-making, and support continuous improvement in areas such as food safety culture maturity and organisational performance.

- Data analytics platforms: Tools to visualise trends and correlations in quantitative data collected from surveys, audits, or inspections.
- AI-Powered survey tools: Platforms to analyse responses, identify anomalies, and generate actionable insights in real-time.
- Predictive analytics: AI systems that use historical data to predict potential food safety risks or trends in FSC, helping to anticipate issues before they occur.
- Machine learning (ML) algorithms: Machine learning models that can identify hidden patterns in large datasets (e.g., employee behaviour, training completion, and incident reports) related to food safety.
- Qualitative coding software: AI-enhanced tools that automate coding of qualitative data to facilitate the categorisation of themes and generate insights about FSC.
- Mobile inspection apps: Applications equipped with AI to assess food safety compliance and generate real-time reports, helping organisations to spot weaknesses in their safety culture.
- Voice-to-text AI: Tools that transcribe interviews, focus groups, and verbal reports into structured data for further analysis, reducing the time needed to process qualitative data.

### 6.5. Sentiment Analysis

**Figure 6:**
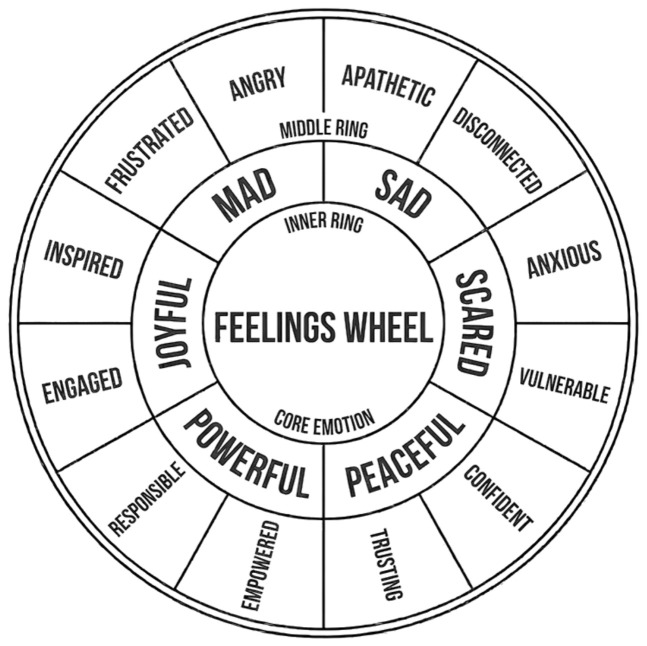
Example of a sentiment/feelings wheel.

Automated sentiment analysis includes tools that apply AI to analyse sentiment from employee feedback to assess the overall FSC perception within a company or organisation. This sentimental data can be presented in narrative format or visualised through feelings wheels (Figure 6). The methodology evaluates the overall emotional tone and attitudes towards the company’s FSC by applying natural language processing (NLP), machine learning techniques, and AI to identify sentiments such as satisfaction, frustration, or disengagement, thereby assisting organisations to assess the health of their culture and propose effective interventions. Such insights enable organisations to gauge cultural health and design targeted interventions [60,114]. Additionally, sentiment analysis can support the identification of food safety constructs by streamlining employee engagement, evaluating leadership alignment, and monitoring workplace well-being. For example, identify signs of stress, dissatisfaction, or burnout in employee communications, allowing them to take proactive steps to improve well-being and foster a positive food safety environment. Compared with manual methods, sentiment analysis boasts additional strengths above manual or less advanced technologies, such as providing real-time insights, identifying underlying issues, ensuring objective measurement and limiting bias, tracking changes over time, ensuring scalability and improving decision-making and aligned interventions [18,60,114].

### 6.6. Exploring Secondary Data

The application of secondary data analysis and meta-analysis in the assessment and improvement of FSC has been increasing in recent years. This trend reflects the increasing need for benchmarking and the strategic use of data collected through commissioned and advisory interventions [116]. These methodologies offer several benefits [34,68,116,117], not only in terms of food safety, but also in the fields of academia, research and postgraduate studies.

#### 6.6.1. Secondary Data

- Cost-effective: Utilising existing data (e.g., previous surveys, audits, and inspection reports) eliminates the need for new data collection, saving time and resources.
- Broader perspective: Secondary data allows for a broader view of FSC across multiple locations or organisations, helping identify patterns or trends that might not be obvious in a single dataset.
- Historical insights: Analysis of historical data provides insight into how FSC changed over time and the effectiveness of past interventions.
- Contextual comparison: Secondary data can be used to compare food safety practices and culture across different sectors or regions, helping organisations benchmark their own culture against industry standards.

#### 6.6.2. Meta-Analysis

- Aggregated insights: Meta-analysis combines results from multiple studies or data sources, providing a more robust and comprehensive view of FSC trends and influencing factors.
- Increased statistical power: By combining data from several studies, meta-analysis increases the sample size, leading to more reliable and generalised conclusions about FSC.
- Identifying common factors: This meta-analysis method helps pinpoint common factors that contribute to successful FSC improvements, which can guide best practices for broader application.
- Improved decision-making: Meta-analysis enables organisations to base decisions on a larger body of evidence, increasing the likelihood of implementing effective FSC initiatives.

### 6.7. Interplays Between Food Safety, Quality, and Occupational Hygiene and Safety (OHS)

FSC focuses on creating an organisational environment that prioritises the prevention of foodborne illnesses and ensures compliance with health regulations through safe practices, hygiene, and risk management [20,49,60]. It emphasises adherence to standards, protocols, and continuous monitoring to safeguard public health. In contrast, food quality culture centres around producing products that meet or exceed customer expectations for taste, appearance, texture, and consistency, fostering a commitment to excellence in every aspect of production. While distinct in their core objectives, these two cultures are closely interrelated. Both cultures share a focus on high standards. FSC emphasises risk mitigation and regulatory compliance, whereas food quality culture stresses product consistency and customer satisfaction. Together, they form a synergistic foundation for overall food system resilience, regulatory compliance, and consumer trust [30,32,59].

While they serve different primary goals, one protecting the consumer and the other protecting the worker, food safety and occupational health and safety (OHS) are deeply intertwined, with FSC having much of its roots in OHS-informed organisational culture. Also, the internal food safety portfolios of food organisations are often also responsible for OHS, with the broader staff component often failing to differentiate between the two. Fortunately, the controls put in place to manage biological, chemical, and physical hazards inherently offer benefits to employees and food handlers. For instance, robust ventilation systems eliminate airborne contaminants that could spoil food while simultaneously protecting employees from inhaling toxic fumes or dust. Similarly, strict handwashing protocols, mandatory personal protective equipment (PPE), and rigorous chemical sanitisation routines guard workers against workplace infections and toxic exposures while maintaining the sterile, pathogen-free environment required for uncontaminated food production. Ultimately, a failure in one is likely to compromise the other.

## 7. Conclusions

FSC remains a novel concept that has created a lot of enthusiasm, but also some turmoil in the food sector, primarily due to uncertainty about its implementation, standardisation, and assessment. The narrative that food safety should be a priority at all levels rather than an isolated, delegated function has also seen some push ack from leadership and peripheral organisational portfolios that regard food safety as the “the blue-eyed boy” to be elevated above other portfolios in the production chain. Nevertheless, in recent years, FSC’s importance has been increasingly recognised, shifting the focus from compliance-based approaches to proactive, people-centred strategies. The integration of behavioural science and digital tools, parallel to the usual biological perspectives to measure and enhance safety culture, has rendered the concept novel, dynamic, and responsive. Both opportunities and challenges remain with the FSC protocols and interventions, particularly in matters such as assessment standardisation across diverse global supply chains and ensuring that all employees, across organisational levels and protocols, are engaged and committed. Ultimately, the intent of the standards guiding FSC is to ensure continuous improvement through initiatives that embrace new and innovative technologies, rather than remaining stagnant [118]. Looking ahead, opportunities lie in leveraging novel data analytics, AI protocols, and novel ethical and interdisciplinary systems that foster transparency and resilience, which not only mitigate risks but also enhance organisational well-being and productivity, and build consumer trust in the integrity of the farm-to-food continuum.

## Figures and Tables

**Table 1:** The evolution of the FSC standard.

Year of Origin	Different Principles Through the Evolution
1960s	HACCP principles created (later published as Dutch-GFSI approved)
1969	Codex Alimentarius published general principles of food hygiene
1991	HACCP became part of EU regulation
1996	HACCP standard published in South Africa
2005	ISO 22000 first issued
2009	ISO 22000 became part of the FSSC 22000 certification scheme
2018	ISO 22000:2018 second edition published
2023	Later version of FSSC 22000 scheme certification published (v6.0)

HACCP, Hazard Analysis and Critical Control Points; GSFI, Global Food Safety Initiative; ISO, International Organisation for Standardisation; EU, European Union; FSSC, Food Safety System Certification.

**Table 2:** Science vs. behaviour-based approaches to food safety.

Features	Scientific-Based Food Safety Management	Behaviour-Based Food Safety Management
Primary focus	Emphasis on adherence to scientific principles, regulations, and standards.	Focuses on shaping employee behaviour, attitudes, and decision-making relating to food safety.
Approach to risks	Identifies and manages risks based on scientific analysis, testing, and data.	Acknowledges the role of human behaviour in creating and mitigating risks, aiming to influence behaviour to prevent errors.
Regulatory compliance	Primarily driven by compliance with industry regulations and standards.	Incorporates regulatory compliance but places a significant emphasis on influencing employee behaviour to exceed minimum requirements.
Root cause analysis	Utilises scientific methods to investigate and address the root causes of issues.	Integrates behavioural analysis alongside scientific methods for a holistic understanding of root causes, emphasising human factors.
Training	Focuses on technical training to ensure employees understand and follow established protocols.	Includes behavioural training to influence employee attitudes, decision-making, and the adoption of safe practices.
Continuous improvement	Continuous improvement is driven by scientific analysis of processes and outcomes.	Encourages continuous improvement by actively involving employees in identifying areas for enhancement and addressing behavioural aspects affecting food safety.
Responsibility and accountability	Places responsibility on following established procedures and standards.	Encourages a shared responsibility for food safety, emphasising individual and collective accountability for behaviour and decision-making.
Employee involvement	Employees follow established protocols and guidelines.	Actively involves employees in identifying risks, suggesting improvements, and shaping a positive FSC through behavioural contributions.
Feedback and reporting systems	Focuses on data-driven feedback systems based on scientific measurements.	Encourages open communication, reporting of near-misses, and a feedback loop that includes both scientific data and insights into behavioural aspects.
Cultural emphasis	Cultural emphasis on adherence to standards and procedures.	Shifts cultural norms to prioritise a proactive approach to food safety, with a focus on influencing behaviour for a continuous improvement mindset.
Preventive measures	Emphasis on preventive measures derived from scientific analysis.	Extends preventive measures beyond technical aspects, addressing behavioural elements to prevent errors and enhance overall food safety.

**Table 3:** Food safety culture approaches in BRCGS vs. FSSC standards (adopted from www.brcgs.com and www.fssc.com).

Constructs	BRCGS	FSSC 22000
Framework structure	Provides FSC excellence module as part of standards.	Integrates FSC into broader scheme without standalone module.
Integration with existing standards	FSC integrated into global standards for food safety.	Integrates FSC into ISO 22000-based certification.
Flexibility and adaptability	The flexibility and adaptability of the FSC excellence assessment module of BRCGS empower organisations to implement an FSC program that is tailored to their specific context, fostering a more effective and sustainable approach.	FSC is not a separate management system and should be integrated in the FSMSs, and adds the element of culture next to the existing focus on food safety.
Assessment methods	The BRCGS FSC excellence assessment is based on the following structure: PEOPLE: Empowerment, reward, teamwork, training, and communication. PROCESS: Control, coordination, consistency, systems, and premises. PURPOSE: Vision, values, strategy, targets, and metrics. PROACTIVITY: Awareness, foresight, innovation, learning, and investment.	Communication: Clause 4. Context of the organisation: Clause 5. Leadership: Clause 7. Support training: Clause 7.2 Competence: Clause 7.3. Awareness feedback from employees: Clause 5.3. Organisational roles, responsibilities, and authorities: Clause 5.3.2. Organisational roles, responsibilities, and authorities: Clause 7.3. Awareness: Clause 7.5.1. Documented information measurement: Clause 4.
Global recognition	Widely recognised globally, especially in retail and food manufacturing.	Recognised by GFSI, with international respect.

**Table 5:** Methodologies and considerations for survey items’ formulation.

Features	Methods	Description
Define objectives	Clarify goals	Clearly outline the purpose of the questionnaire to guide content development (e.g., measuring attitudes, behaviours, and demographics).
Identify constructs	Conceptual framework	Develop a theoretical framework to identify fundamental constructs (e.g., satisfaction and loyalty) that need to be measured.
Question types	Closed-ended vs. open-ended questions	Use a combination of closed-ended questions (for quantitative data) and open-ended questions (for qualitative insights).
Question wording	Simplicity and clarity	Keep questions clear, concise, and free from ambiguity. Avoid jargon, double-barrelled questions, or leading questions.
Scaling techniques	Likert, semantic differential, and rating scales	Use standardised scales (e.g., Likert scale) for measuring attitudes, opinions, or behaviours on a defined scale.
Question flow	Logical sequence	Organise questions in a logical order: introductory questions first, followed by main content questions, and ending with demographic questions.
Pre-testing	Pilot testing	Conduct a pre-test with a small sample to identify issues with wording, structure, or clarity and refine the questionnaire.
Length and brevity	Concise and focused	Keep the questionnaire short and to the point to maintain respondent engagement and minimise fatigue.
Response options	Mutually exclusive and exhaustive	Provide clear and distinct response options to avoid overlap and ensure all potential answers are covered.
Demographic items	Placement at the end of questionnaire	Ask demographic questions towards the end to avoid biasing responses to core questions and to maintain respondent comfort.
Language and tone	Neutral tone	Ensure the language is neutral and non-leading, avoiding emotional or biased wording that could influence responses.
Validation	Validation and reliability	Use validated scales where possible and ensure consistency by including control questions to test reliability.
Data analysis considerations	Pre-define variables	Identify variables for analysis upfront (e.g., age, income, and satisfaction) to ensure questions align with intended data analysis.

**Table 6:** An example of a data collection questionnaire for food safety culture assessment.

FOOD SAFETY CULTURE SURVEY Kindly state your section/department/division/unit (voluntary): ____________________________ (By completing the survey, you acknowledge participation to be voluntary, anonymous, and not cause any harm; you consent to being informed about the purpose and intent of the survey; and that your participation will not infringe on the protection of personal information).					
INSTRUCTIONS: Indicate your degree of disagreement or agreement with the listed statements on a scale from strongly disagree (0), to disagree (1), neutral (2), agree (3), and, finally, strongly agree (4) by marking with an “X” in the relevant blocks. Answer all questions irrespective of your role; should you opt not to answer, disregard and move to the next.	Strongly disagree	Disagree	Neutral	Agree	Strongly agree
	0	1	2	3	4
VISION: (The existence of clear direction and understanding of food safety goals, expectations, and targets, encouraged by ethical, logical, and compassionate leadership through effective communication and across all occupational levels).					
Food safety is important to our organisation.					
2. Our organisation has capable and approachable leadership.					
3. Our leaders and managers lead by example in terms of food safety.					
4. Food safety is important to all employees and across all levels.					
5. I know and understand our product safety objectives and expectations.					
6. I have an important role to play in our organisation’s food safety performance.					
7. The targets and expectations my company has of us regarding food safety are well communicated.					
8. I am aware of policies and processes at our organisation that guide our food safety and quality commitment.					
9. We often talk about food safety in our organisation and sections.					
10. We are made aware of the importance of food safety though, for example discussions, notices, signage, illustrations, seminars, announcements, and/or social media.					
Optional comments—Please explain and allude to your responses above:					
INSPIRATION: (The confidence and participation of employees on all levels to engage in food safety, facilitated through actions that create a sense of loyalty, ownership, responsibility, and confidence).					
	0	1	2	3	4
I am happy to raise apparent food safety concerns, risks and improvement opportunities.					
2. I am free to question the way things are done and raise opposing views.					
3. I am committed to making inputs and suggestions beyond my main functions.					
4. I am accountable for food safety gaps in my area.					
5. My opinions and inputs are valued and respected.					
6. I care for our customers.					
7. I feel part of food safety and quality decision making.					
8. My organisation and I are loyal towards, and trust each other.					
9. My organisation cares about its people and make them a priority.					
10. We have a high morale and enjoyable team-spirit at work.					
Optional comments—Please explain and allude to your responses above:					
EMPOWERMENT (The provision of, and access to adequate and relevant training and resource provision, including knowledge and skills, infrastructure, equipment, finances, and staff to enable achievement of the organisation’s food safety goals and expectations).					
	0	1	2	3	4
I am eager to obtain and practice new knowledge and skills.					
2. I have the relevant skills to do my work well and with confidence.					
3. I am aware of the risks and dangers associated with food safety.					
4. I know when a particular food safety incident may constitute a substantial risk.					
5. The training we receive is adequate and effective.					
6. I know the difference between, and importance of both food safety and quality.					
7. Our organisation has appropriate induction programs for new employees.					
8. In our organisation, resources to ensure food safety are equally important amongst sections and departments.					
9. My outfits, tools, and materials are adequate to do my job well.					
10. Faulty, defective, or old equipment are replaced and maintained regularly.					
Optional comments—Please explain and allude to your responses above:					
FOOD SAFETY PERFORMANCE (Maintenance of responsive and consistent documented food safety management systems and procedures, which includes relevant compliance and risk assessment instruments, monitoring, metrics, evaluation, consequences, achievements, and improvement measures).					
	0	1	2	3	4
Auditors and inspectors are treated with respect and appreciation.					
2. Compliance policies and protocols are respected and understood.					
3. Our organisation regards regulatory and compliance inspections and audits as important.					
4. Outcomes of inspections and audits are transparent and extensively communicated to staff.					
5. We employ consistent, applicable, and updated food safety management systems.					
6. We keep proper and updated records of food safety processes and compliance.					
7. Our organisation takes customer complaints relating to both product quality and safety seriously.					
8. We widely celebrate and reward our food safety successes and achievements.					
9. Our company is recognised and acknowledged for exceptional product safety and quality.					
10. I am mindful of rewards and consequences relating to food safety and quality performance outcomes.					
Optional comments—Please explain and allude to your responses above:					
CHANGE IN APPETITE AND IMPROVEMENT (Our organisation’s level of awareness, respect, and equitability across portfolios relating to culture, race, gender, and levels of authority, and its agility and progressiveness to change and improve in response to emerging technological, economical, societal, and organisational demands that may influence food safety performance).					
	0	1	2	3	4
I believe that change and improvement will be beneficial to our food safety approach.					
2. I trust our leaders to have foresight, and manage change processes responsibly, effectively and fairly.					
3. I am informed about future plans and strategies.					
4. I am involved, and take part in change and improvement processes.					
5. Our organisation acknowledges and supports diversity, equality, cultures, and customs.					
6. Race, gender, political and religious orientation plays no role in how I am treated and acknowledged as a food safety and quality champion.					
7. I see our organisation as visionary and dynamic rather than old-fashioned and stagnant.					
8. All employees are treated equally in terms of opportunities, recognition, and consequences.					
9. Our organisation embraces new technologies and innovations.					
10. I see myself continuously developing and achieving my potential at this organisation.					
Optional comments—Please explain and allude to your responses above:					
INTERVENTION PREFERENCES (For FSC improvement, select interventions that would be accepted and effective in your organisation. You may select multiple options).					
	0	1	2	3	4
Training.					
2. Practical demonstrations and role-play.					
3. Food safety champions and ambassadors.					
4. Team building and social events.					
5. Recognition and acknowledgement programs.					
6. Inspirational messaging and branding.					
7. Effective and transparent communication.					
8. Leadership visibility and involvement.					
9. Resources to simplify and improve my work.					
10. Rewards and incentives.					
11. Competitions.					
12. Work–life sensitivity and wellness programs.					
13. Effective reporting and whistle blowing procedures.					
Other/Optional comments and suggestions:					

**Table 7:** An example of an agenda for FSC focus group discussions.

Focus Group Agenda: Assessing Food Safety Culture
Objective To collect qualitative insights into employees’ perceptions, attitudes, and behaviours around food safety practices, leadership commitment, and overall FSC within the facility.
*A. Welcoming and Introductions (10–15 min)*
1. Facilitator Introduction:
• Briefly introduce yourself, your role, and the purpose of the session.
• Explain the confidentiality and ethical considerations (e.g., no right or wrong answers, and anonymity of responses).
• Outline the structure and timing of the session.
• Participant Introductions:
• Ask participants to introduce themselves (name, role, how long they have worked at the company, and their general experience with food safety practices).
2. Icebreaker/Opening question (5–10 min)
Opening Question:
• “To get started, can you each share one thing that you think is most important for ensuring food safety in the workplace?”
• This will help warm up the group, get them thinking about food safety, and set the tone for the discussion.
3. Discussion of Core Topics (45–60 min)
*B. Awareness and perception of food safety culture (15 min)*
• Question 1: “How would you describe the food safety culture here? What does food safety mean to you personally?”
• Question 2: “In your opinion, how strongly does leadership emphasise the importance of food safety? Can you give examples?”
• Question 3: “Do you feel that food safety is a priority for the company as a whole, or is it seen as secondary to other tasks?”
*C. Training, communication, and knowledge (10–15 min)*
• Question 4: “What kind of training or resources have you received on food safety, and how effective do you think these have been in preparing you for your role?”
• Question 5: “How do you receive information about food safety updates or changes in procedures? Is communication clear and consistent?”
• Question 6: “Can you share any examples where you felt confident or unsure about food safety practices because of the training or communication you received?”
*D. Food safety practices and employee behaviour (10–15 min)*
• Question 7: “What are some of the most common food safety practices you follow daily? Are there any practices that are hard to maintain consistently?”
• Question 8: “How do your coworkers approach food safety? Do you notice any differences in behaviours, and how does that affect the work environment?”
• Question 9: “How do you feel when you see a colleague not following food safety procedures? What actions do you take in those situations?”
*E. Challenges and barriers to food safety (10–15 min)*
• Question 10: “What are some challenges you face in maintaining food safety standards in your role?”
• Question 11: “Are there any areas where you feel the company could improve its food safety culture or practices?”
• Question 12: “Have you ever encountered situations where food safety policies or procedures were not followed? How was that handled, and what was the impact on the work environment?”
*F. Discussion and Final thoughts (10–15 min)*
• Question 13: “If you had the power to change one thing about the food safety culture here, what would it be and why?”
• Question 14: “What additional support or resources would help you feel more confident in following food safety practices?”
*G. Closing remarks:*
• Thank the participants for their time and input.
• Reiterate how their feedback will be used and the importance of their insights in improving food safety culture.
• Next steps: Inform participants about any follow-up actions (e.g., summary report, additional surveys, and next round of discussions).
• Total Time: 90 to 120 min.
Facilitator suggestions:
• Reiterate voluntary participation, anonymity, and confidentiality and enable participants not opting to participate due to possible interpersonal dynamics with fellow focus group participants to register their feedback in writing, or the option to participate in the survey and thereby recording both qualitative and quantitative responses • Encourage free dialogue: Ensure that everyone has a chance to speak and encourage participants to share their experiences and views. • Probe for details: Ask follow-up questions to clarify and probe deeper into participant responses. For example: “Can you tell me more about that?” or “Why do you think that is?” • Manage group dynamics: Ensure no single participant dominates the conversation and try to draw out quieter participants. • Keep the discussion focused: If the conversation strays too far from the core topics, gently steer it back to the central themes related to food safety culture. • Utilise the services of a scribe or record to ensure effective data capturing

**Table 8:** An example of an FSC walk-through checklist.

Facility Walk-Through Checklist: Assessing Food Safety Culture
1. Leadership commitment
• Visible leadership support: Are food safety goals and policies prominently displayed? Is there evidence that leadership supports food safety practices (e.g., managers participating in food safety training)?
• Communication from leadership: Does leadership communicate the importance of food safety regularly (e.g., during meetings, through memos, or newsletters)?
• Resource allocation: Are adequate resources (time, personnel, and equipment) allocated to food safety initiatives and training?
2. Employee engagement and behaviour
• Employee training: Are employees regularly trained on food safety practices, and is there documentation of training? Is training ongoing or periodic?
• Employee accountability: Do employees take responsibility for maintaining food safety standards (e.g., proper handwashing and cleaning procedures)?
• Reporting mechanisms: Are employees encouraged to report food safety concerns or violations without fear of retribution? Is there a system for reporting incidents?
• Food safety as part of culture: Do employees seem aware of and committed to food safety standards in their daily actions (e.g., wearing PPE and following hygiene protocols)?
3. Hygiene and sanitation practices
• Personal hygiene: Are employees following proper handwashing and personal hygiene procedures (e.g., use of gloves, hairnets, and handwashing stations)?
• Sanitation stations: Are handwashing stations and sanitising stations fully stocked, accessible, and in good condition?
• Cleaning schedules: Are cleaning schedules posted, and is there evidence that they are being followed (e.g., clean workstations, equipment, and restrooms)?
• Cleanliness of equipment: Is food contact equipment (e.g., cutting boards, and knives) visibly clean? Are there signs of contamination or build-up?
4. Food safety practices and compliance
• Temperature control: Are food storage and preparation areas maintained at the correct temperatures (e.g., refrigerators, freezers, and hot-holding units)?
• Cross-contamination prevention: Are proper separation practices followed to avoid cross-contamination (e.g., raw vs. cooked foods, allergens, and separate utensils)?
• Pest control: Are there any signs of pests (e.g., rodents or insects)? Are pest control measures visibly in place and effective?
• Food safety documentation: Are critical food safety documents (e.g., HACCP plans, temperature logs, and cleaning records) readily accessible and up to date?
5. Equipment and facility maintenance
• Facility conditions: Is the physical condition of the facility in good order (e.g., no leaks, cracks, or standing water)? Are floors, walls, and ceilings well-maintained?
• Equipment maintenance: Is food processing equipment regularly maintained and serviced? Are maintenance records up to date?
• Proper lighting: Are areas well-lit, especially those where food is prepared or stored, to ensure thorough inspection and cleanliness?
• Adequate ventilation: Are ventilation systems functional, ensuring that air flow in the facility is clean and free from contaminants?
6. Food handling and storage
• Food storage: Are food products properly stored (e.g., correct temperatures, no food items on the floor, and proper separation of food categories)?
• Food labelling: Are food items clearly labelled with expiration dates and handling instructions? Is inventory rotated (e.g., FIFO—first in, first out)?
• Handling practices: Are food handling practices consistent with safe food handling guidelines (e.g., no bare-hand contact, proper use of utensils, etc.)?
7. Emergency preparedness and incident response
• Emergency plans: Are food safety emergencies (e.g., contamination and recall) addressed in clear procedures? Is the plan visible to employees?
• Recall procedures: Are recall procedures in place and understood by employees in case of a product recall? Is there evidence of practice or drills?
• Response to violations: Are there clear actions taken when food safety violations occur (e.g., corrective actions, retraining, and retracing food products)?
8. Continuous improvement
• Regular audits: Are internal or external food safety audits conducted regularly, and are corrective actions documented and implemented?
• Employee feedback: Is there a process for gathering and acting on employee feedback related to food safety practices?
• Monitoring systems: Are food safety performance metrics (e.g., audit scores or incident reports) being monitored and used for continuous improvement?
9. Compliance with regulations and standards
• Regulatory compliance: Does the facility comply with local, state, and federal food safety regulations (e.g., FDA or USDA)? Are there signs of recent inspections?
• Certifications: Is the facility certified by recognised food safety standards (e.g., ISO 22000, SQF, or BRC)? Are certificates displayed?
10. Final assessment
• Overall impression of food safety culture: Is there a visible commitment to food safety across all levels of the organisation? Does the facility exhibit a proactive rather than reactive approach to food safety?
• Areas of concern/improvement: List specific areas where food safety culture could be improved, e.g., more frequent employee training, better sanitation practices, etc.

**Table 11:** A cognitive progression model for the interpretation of FSC assessment data [107].

Step	Element	Verb Level 1	Verb Level 2
1	Explain	Visualise	Generate, create, calculate, augment, repeat, expand.
		Organise	Analyse, separate, group, optimise, categorise, arrange, order, articulate.
		Summarise	Delineate, simplify, prioritise, select, restrict, omit, flow.
2	Discuss	Elaborate	Differentiate, classify, distinguish, identify, verify, assess.
		Comprehend	Synthesise, compare, integrate, understand, realise, ideate, innovate.
		Evaluate	Appraise, judge, value, critique, contradict, discriminate, dispute, defend, challenge.
3	Conclude	Recapitulate	Acknowledge, focus, prioritise, outline, recap, revisit, reconsider, reiterate.
		Apply	Solve, align, extrapolate, relate, execute, perform, amend, improve, adjust, fix, establish, ideate, test.
		Recommend	Inform, present, suggest, propose, establish, promote, endorse, facilitate, collaborate, prospect, advise.

**Table 12:** FSC maturity calculation per construct (illustrative data).

	Strongly Disagree	Disagree	Neutral	Agree	Strongly Agree	SUM Weight/Question ^b^	Score/ Question % ^c^	Weighted Contribution/Question ^d^
WEIGHT	0	1	2	3	4			
I know and understand our food safety policies and goals.	0	1	3	19	27	172	86.0	17.2
2. I know what is expected from me regarding food safety.	0	1	1	16	32	179	89.5	17.9
3. I do not tolerate food safety lapses in my area.	1	0	5	13	31	173	86.5	17.3
4. I have the knowledge and tools to do my work well.	2	4	5	16	25	162	81.0	16.2
5. I have right food safety skills to do my job well.	1	4	5	14	26	160	80.0	16.0
SUM ^a^	4	10	19	78	141	846	846 per 1000	MATURITY SCORE (%) ^f^ 84.6%
SUM Weight (Total of responses × weight) ^e^	0	10	38	234	564			

*N* = 50; questions = 5; minimum grade/question: 0 (Weight) × 50 (Respondents) = 0; maximum grade per question: 4 (Weight) × 50 (Respondents) = 200; total weight per category = 4 (Weight) × 5 (Questions) × 50 (Respondents); score/question = SUM weight per Likert category × number of respondents across all categories. ^a^: visualises the performance of the organisation in terms of the specified construct (e.g., vision). Used in conjunction with the maturity score; ^b,c^: used predominantly in graphs to indicate the scores of the individual questions relative to each other and also used to identify particular outliers; ^d^: indicates the scores in accordance with weighting—not used often; ^e^: also used to indicate the performance of the organisation in terms of the specified construct, but mostly for the maturity score calculation; and ^f^: used to indicate the maturity and performance of constructs against one another at a glance (builds upon a. if required). The above calculations and results can be used randomly to assist in explaining findings and drawing conclusions.

**Table 14:** Phase 3 of the cognitive progression model, advancing to competencies such as conceptualising multidisciplinary and multi-faceted solutions that are applied, achievable and aligned with standards and audit expectations.

Conclude	Recapitulate	Acknowledge, focus, prioritise, outline, recap, revisit, reconsider, reiterate.
	Apply	Solve, align, extrapolate, relate, execute, perform, amend, continuously improve, adjust, fix, establish, ideate, test.
	Recommend	Inform, present, suggest, propose, establish, promote, endorse, facilitate, collaborate, prospect, advise.

**Table 15:** Summary of the principles and benefits of peer and expert review.

1. Enhanced credibility and validity
Validation of findings: Experts, especially with proven experience in the food industry, bring domain-specific knowledge and experience that can help validate the findings from different data sources (e.g., surveys, interviews, and observations). Their input can confirm whether the triangulated data aligns with existing theories, practices, or empirical evidence.
Cross-checking data sources: Since triangulation involves using various data sources (qualitative and quantitative), expert review may assist in ensuring that the conclusions drawn from the data are consistent and reliable. They can flag any inconsistencies or contradictions between different data types and help reconcile these discrepancies.
2. Informed interpretation
Contextualising data: Organisational culture data often requires contextual interpretation to make sense of complex social phenomena. Experts can provide insights into the social, cultural, political, or historical context that may not be immediately apparent from the data alone.
Knowledge of the food safety field: Experts can draw on their theoretical and practical knowledge of the field to offer a deeper interpretation of the results, particularly when the data reflects complex or unfamiliar issues. Their expertise can highlight nuances in the data that might be overlooked by non-experts or those less familiar with the subject matter.
3. Identification of biases and blind spots
Reducing bias: Assessors or internal quality and safety assurance staff may bring their own biases or preconceptions to the interpretation of data. By involving a diverse panel of experts, the potential for researcher bias can be reduced. Experts can provide an external perspective and identify areas where the interpretation may be skewed or incomplete.
Multiple perspectives: Expert panels typically consist of individuals with different areas of expertise, which can prevent narrow interpretations of the data. This diversity of thought helps to ensure that the findings are not limited to one perspective or framework.
4. Improved quality of analysis
Methodological rigor: Experts can ensure that the triangulation process is conducted correctly, and that the data is analysed using appropriate techniques. They can offer advice on the best methods for integrating different types of data (qualitative and quantitative) or suggest alternative approaches to interpretation when necessary.
Addressing complexity: Social research often involves complex, multifaceted issues. Expert panel review helps break down these complexities and ensures that the interpretation reflects the full scope of the data, including any contradictions, uncertainties, or underlying patterns.
5. Increased trustworthiness of conclusions
Peer review: Expert panel review acts as a form of peer review, ensuring that the findings and interpretations are subjected to scrutiny by knowledgeable professionals. This process can increase the trustworthiness of the conclusions drawn from triangulated data, particularly when the study’s conclusions are used for policy decisions, advocacy, or further investigation.
Accountability: The expert panel adds a layer of accountability to the FSC improvement process, and also strengthens the evidence presented during audits. By critically evaluating the interpretation of triangulated data, the panel ensures that the study’s findings are not just the product of the researchers’ biases or limited perspectives but are instead grounded in sound, multi-faceted analysis.
6. Identification of patterns and relationships
Synthesising complex data: When triangulating different data sources, it can sometimes be difficult to draw meaningful connections between disparate datasets. Experts can synthesise the data across sources and methods, identifying patterns, relationships, or emerging trends that might not be immediately obvious.
Generating insights: Experts can offer insights into new directions for future FSC interventions based on the triangulated data. They might spot gaps or inconsistencies that suggest the need for further exploration or propose new questions that were not initially considered.
7. Cross-Disciplinary acumens
Holistic view: In FSC surveys, issues are often complex and require insights from multiple disciplines. An expert panel with diverse disciplinary backgrounds can provide a holistic view of the data, ensuring that all relevant aspects of the social issue are considered.
Theory and practice integration: Experts who have both academic and practical experience can integrate theoretical frameworks with real-world application, making the interpretation of the data more grounded in both evidence and practice.
8. Mitigation of interpretation errors
Disagreement and debate: In cases where the triangulated data is ambiguous or when there are differing interpretations, expert panel members can engage in discussion and debate to arrive at a more balanced, consensus-based interpretation. This collaborative approach helps reduce the likelihood of erroneous or overly simplistic interpretations.
Clarifying ambiguities: Social data can sometimes be ambiguous, and different stakeholders may interpret it differently. Experts can help clarify these ambiguities by providing additional insights or suggesting ways to refine the analysis.
9. Ethical review and social impact considerations
Ethical evaluation: Experts can ensure that the interpretation of data is ethically sound, especially when the data involves sensitive issues (e.g., marginalised groups, vulnerable populations, or controversial social issues). They can also advise on how to interpret the data in a way that is socially responsible and that minimises potential harm.
Policy implications: When triangulated data is used to inform policy decisions, expert panels can help assess the broader social implications of the findings. They can ensure that the interpretations are not only scientifically valid but also socially relevant and appropriate for the intended audience.
10. Increased transparency and rigor
Justification of findings: By involving a panel of experts, the study’s findings and interpretations are better justified. Expert review provides an additional layer of transparency to the FSC assessment process, which is crucial for building trust in the study’s conclusions.
Documentation of decisions: The panel’s input helps document the rationale behind decisions in the interpretation process. This documentation can be valuable for auditing purposes, future leadership and stakeholders who seek to understand how triangulated data was handled and interpreted in a particular study.

**Table 16:** Distinctions between FSMSs and FSC auditing [49].

Features	FSMS Auditing	Food Safety Culture Auditing
Focus	Processes, procedures, and compliance.	Attitudes, behaviours, and leadership.
Methodology	Document-based and compliance-oriented.	Behavioural, qualitative, and observational.
Evidence	Records, policies, and procedures.	Employee perceptions, behaviours, and feedback.
Objective	Ensure compliance with food safety standards.	Assess and improve food safety attitudes and behaviours.
Scope	Focused on technical aspects of food safety systems.	Broader organisational culture and engagement.
Frequency	Scheduled audits (annually or per standards).	Continuous monitoring and ongoing feedback.
Auditor Expertise	Food safety and regulatory compliance experts.	Behavioural science, human resources, and organisational culture experts.

## Data Availability

No new data were created or analysed in this study. Data sharing is not applicable to this article.
